# Oxidative potential of the inhalation bioaccessible fraction of PM_10_ and bioaccessible concentrations of polycyclic aromatic hydrocarbons and metal(oid)s in PM_10_

**DOI:** 10.1007/s11356-024-33331-9

**Published:** 2024-04-19

**Authors:** Natalia Novo–Quiza, Joel Sánchez–Piñero, Jorge Moreda–Piñeiro, Isabel Turnes-Carou, Soledad Muniategui–Lorenzo, Purificación López–Mahía

**Affiliations:** Department of Chemistry, Faculty of Sciences, Grupo Química Analítica Aplicada (QANAP), University Institute of Research in Environmental Studies (IUMA), University of A Coruña, Campus de A Coruña, S/N. 15071, A Coruña, Spain

**Keywords:** Oxidative potential, Physiologically based extraction, Gamble solution, Bioaccessible fraction, Dithiothreitol, Ascorbic acid assays

## Abstract

Atmospheric particulate matter (PM) has been related to numerous adverse health effects in humans. Nowadays, it is believed that one of the possible mechanisms of toxicity could be the oxidative stress, which involves the development of reactive oxygen species (ROS). Different assays have been proposed to characterize oxidative stress, such as dithiothreitol (DTT) and ascorbic acid (AA) acellular assays (OP^DTT^ and OP^AA^), as a metric more relevant than PM mass measurement for PM toxicity. This study evaluates the OP of the bioaccessible fraction of 65 PM_10_ samples collected at an Atlantic Coastal European urban site using DTT and AA assays. A physiologically based extraction (PBET) using Gamble’s solution (GS) as a simulated lung fluid (SLF) was used for the assessment of the bioaccessible fraction of PM_10_. The use of the bioaccessible fraction, instead of the fraction assessed using conventional phosphate buffer and ultrasounds assisted extraction (UAE), was compared for OP assessment. Correlations between OP^DTT^ and OP^AA^, as well as total and bioaccessible concentrations of polycyclic aromatic hydrocarbons (PAHs) and metal(oid)s, were investigated to explore the association between those compounds and OP. A correlation was found between both OP (OP^DTT^ and OP^AA^) and total and bioaccessible concentrations of PAHs and several metal(oid)s such as As, Bi, Cd, Cu, Ni, and V. Additionally, OP^DTT^ was found to be related to the level of K^+^.

**Supplementary Information:**

The online version contains supplementary material available at 10.1007/s11356-024-33331-9.

## Introduction

Airborne particulate matter (PM) is linked to adverse health consequences, including respiratory issues, cardiovascular diseases, and difficulties in neurodevelopmental functions (MacNee 2001; Davidson et al. 2005; Bates et al. 2019; Chen et al. 2022; Zhang et al. 2023). The negative health effects of PM not only depend on the quantity of particles but also on their composition. PM is mostly constituted of low-toxicity components, although several major (chlorides, nitrates, etc.) and minority (transition metals and some organics) compounds have a major impact on PM toxicity (Mudway et al. 2004; Calas et al. 2017; Cigánková et al. 2021). Many inhalable particles are deposited in the respiratory area, and after deposition, particle–bound pollutants interact with the extracellular pulmonary fluids. Pollutants could be dissolved in these fluids and cross the air–blood or alveolar barrier, reaching the circulatory system and posing a health risk. For this reason, the tendency in inhalation risk assessment approaches of PM-associated pollutants has been changing from total contaminant levels to the maximum fraction assessment that could be leached in extracellular pulmonary fluids (bioaccessible fraction) by using in vitro approaches. These approaches simulate the dissolution processes of contaminants using synthetic pulmonary fluids (Kastury et al. 2017; Innes et al. 2021).

In addition to the health risk associated with the pollutant fraction that reaches the bloodstream, the content of pollutants in PM deposited and/or dissolved in pulmonary fluids can induce inflammation and oxidative stress. Inhaled particles produce oxidative stress by transporting reactive oxygen species (ROS) bound to particles into the lungs or by inducing ROS formation by redox-active particle components (Cigánková et al. 2021). Thus, cytotoxicity, genotoxicity, and pro-inflammatory cell responses that PM and contaminants in PM can produce should not be discarded in order to establish an accurate assessment of the potential health risk. In this context, oxidative potential (OP), defined as a measurement of the ability of PM and PM-bound pollutants to deplete certain antioxidant molecules in synthetic fluids (Ayres et al. 2008), has been included in current epidemiological researches, showing relations of OP with numerous health consequences such as asthma and heart failure, instead of total PM mass concentration (Donaldson et al. 2001; Delfino et al. 2013; Bates et al. 2015, 2019; Yang et al. 2016; Rao et al. 2020; He et al. 2021).

Several cell-based and acellular (chemical) assays have been extensively applied to assess the OP of PM. Cell-based assays investigate the production of ROS in animal (mainly rat, murine, and porcine) macrophage cell lines and transformed human bronchial epithelial cell lines that mimic the oxidative stress response of primary epithelial cells (Peixoto et al. 2017; Wang et al. 2018; Øvrevik 2019; He and Zhang 2023). Additionally, acellular assays that directly measure ROS (such as electron spin resonance (ESR), which measures the generation of hydroxyl radical (OH•) in the presence of H_2_O_2_ using spectrometry) and acellular assays that indirectly measure ROS (such as the oxidation of dithiothreitol (DTT), ascorbic acid (AA), urate (UA), total glutathione and oxidized glutathione (GSSG)) have been developed and applied for the assessment of OP-PM (Bates et al. 2019; Jiang et al. 2019; Pietrogrande et al. 2019; Øvrevik 2019; Gao et al. 2020; Rao et al. 2020; Khoshnamvand et al. 2020; Liu and Chan 2022; Shahpoury et al. 2022; Carlino et al. 2023; He and Zhang 2023). However, there is still no clear consensus on the advantages, limitations, and applicability of these assays (Ayres et al. 2008).

Nowadays, acellular assays allow for fast, user–friendly, and less resource-intensive assessments (using inexpensive tools) compared to cellular assays (Bates et al. 2019; Gao et al. 2020). The DTT and AA assays are the most commonly cell-free assays for the assessment of OP in PM samples. Both methods are based on the redox and catalytic capacity of active components of PM to oxidize the reagents (DTT and AA) and the determination of OP as the rate of reagent reduction (OP^DTT^ and OP^AA^), quantified by spectrophotometric techniques. In the DTT assay, DTT is used as a surrogate of nicotinamide adenine dinucleotide phosphate (NADPH), interacting with several PM constituents and producing superoxide radicals. This assay is carried out in two steps. Firstly, DTT is oxidized by redox–active species of PM, generating stable cyclic disulphides that donate electrons to oxygen, forming superoxide ions that can produce hydrogen peroxide and oxygen. Then, the remaining DTT reacts over time using 5.5′–dithiobis(2–nitrobenzoic acid (DTNB), forming DTT–disulphide and 2–nitro–5–thiobenzoic acid (TNB). Due to the strong absorbance of TNB in the visible region, it can be quantified by UV/VIS spectrophotometry (Godri et al. 2010). The depletion of DTT caused by the transference of electrons from DTT to oxygen will be directly proportional to the level of redox-active compounds (including metal(oid)s and highly oxidized organics such as polycyclic aromatic hydrocarbons (PAHs), quinones, secondary organic aerosol, and humic-like substances) in the PM (Cho et al. 2005; Charrier and Anastasio 2012; Gao et al. 2020).

In the second assay, redox and catalytic capacity of the PM are reduced by transferring an electron to oxygen molecules, producing ROS, while the AA is oxidized to dehydroascorbic acid (Godri et al. 2010). Due to the fact that the optical density at 265 nm is exclusive to AA, and since AA reduces exponentially, a linear relationship between the concentration of redox-active compounds (mainly metal(oid)s such as Fe and Cu) in PM and the reduction of AA concentration can be established (Bates et al. 2019).

OP^DTT^ and OP^AA^ have been extensively assessed after PM_10_ extraction by vortex/rotating/shaking (Calas et al. 2017; Massimi et al. 2020; Frezzini et al. 2022a, 2022b; Altuwayjiri et al. 2022; Fang et al. 2023) or ultrasound (Janssen et al. 2014; Perrone et al. 2016; Chirizzi et al. 2017; Rezaei et al. 2018; Patel and Rastogi 2018; Pietrogrande et al. 2018a, b; Perrone et al. 2019; Lionetto et al. 2019; Pietrogrande et al. 2021; Lionetto et al. 2021; Wang et al. 2018; Pietrogrande et al. 2022a, b; Giannossa et al. 2022; Frezzini et al. 2022b Farahani et al. 2022; Molina et al. 2023; Guascito et al. 2023; Clemente et al. 2023) assisted using ultrapure water, methanol, or buffer as solvents 1). However, ultrapure water, methanol, or buffers differ in several characteristics (pH, ionic strength, or the deficiency of complexing ligands) from lung fluids (Calas et al. 2017). On the other hand, the use of ultrasound has been shown to induce the formation of ROS in the solution, enhancing the solubilization of PM insoluble species and causing an overestimation of the OP due to the high efficiency of the extraction assisted by ultrasound energy (Massimi et al. 2020; Frezzini et al. 2022a).

**Table 1:** OP^DTT^ and OP^AA^ (nmol min^−1^ m^−3^) values in PM_10_ samples, including extraction conditions, sampling period, and sample number

OP^DTT^ (nmol min^−1^ m^−3^)	OP^AA^ (nmol min^−1^ m^−3^)	Extraction phases/extraction method	Sampling period/sample number	Sampling site	Reference
0.34 ± 0.16	0.91 ± 0.51	DIW/Sonication	Dec 2020–Aug 2021/60	Urban (Elche; Spain)	Clemente et al. (2023)
0.10–0.50	-	DIW/Vortex mixer	Jan–Dec 2020/8	Urban (California; USA)	Fang et al. (2023)
0.4	-	DIW/Sonication	Nov 2016–Nov 2017/124	Urban (Lecce; Italy)	Giannossa et al. (2022)
0.24–0.3	-	DIW/Sonication	Feb 2019–Jun 2020/116	Urban (Lecce and Aradeo; Italy)	Guascito et al. (2023)
0.24–0.80	-	DIW/Sonication	Jun 2018–Oct 2019/221	Urban (Santiago de Chile and Chillán; Chile)	Molina et al. (2023)
1.2 ± 0.1	-	DIW/Shaking	Dec 2019–Aug 2020/^−b^	Urban (Riyadh; Saudi Arabia)	Altuwayjiri et al. (2022)
1.05–1.85	-	DIW/Sonication	Dec 2019–Aug 2020/63	Urban (Riyadh; Saudi Arabia)	Farahani et al. (2022)
0.0011–1.2	0.04–1.5	DIW/Rotating agitation	May 2021/40	Urban (Terni; Italy)	Frezzini et al. (2022a)
0.22–1.4	0.42–3.9	HNO_3_/Microwave oven	May 2021/40	Urban (Terni; Italy)	Frezzini et al. (2022a)
0.16–3.1	0.18–15	DIW/Sonication or vortex or rotating agitation	Jul 2019–Mar 2020/250	Urban (Rome; Italy)	Frezzini et al. (2022b)
0.39 ± 0.23	-	DIW/Sonication	Nov 2016–Nov 2017/124	Urban (Lecce; Italy)	Giannossa et al. (2022)
0.18–0.72	1.05–2.22	Phosphate buffer (0.1 M at pH 7.4)/Sonication	Apr 2019–May 2020/168	Urban (Milan; Italy)	Pietrogrande et al. (2022b)
0.10–0.39	0.37–2.96	Phosphate buffer (0.1 M at pH 7.4)/Sonication	Jan–May 2020/^−b^	Urban (Milan; Italy)	Pietrogrande et al. (2022b)
1.5 ± 0.5^a^	-	Dichloromethane: Methanol (1:1 V/V)/Sonication	Jan 2016–Jan 2017/44	Urban (Bangkok; Thailand)	Wang et al. (2018)
0.1–0.7	-	DIW/Sonication	Feb–Apr 2019/90	Urban (Lecce; Italy)	Lionetto et al. (2021)
0.33 ± 0.07	0.28 ± 0.08	Phosphate buffer (0.1 M at pH 7.4)/Sonication	Dec 2016–Jan 2017/47	Rural (Novaledo; Italy)	Pietrogrande et al. (2021)
-	-	DIW/Rotating agitation	Sep 2017–Jan 2018/69	Urban (Terni; Italy)	Massimi et al. (2020)
0.15–0.45	-	DIW/Sonication	Jan–Dec 2017/10	Urban (Lecce; Italy)	Lionetto et al. (2019)
0.22 ± 0.02–0.24 ± 0.04	0.23 ± 0.04–0.35 ± 0.06	DIW/Sonication	Dec 2014–Oct 2015/39	Suburban (Salento´s peninsula; Italy)	Perrone et al. (2019)
0.60 ± 0.23	0.7 ± 0.4–1.4 ± 1.1	DIW/Sonication	Feb 2015–May 2016/183	Industrial and background (Alpine region; Italy)	Pietrogrande et al. (2018a)
0.24 ± 0.12	0.29 ± 0.18	Phosphate buffer (0.1 M at pH 7.4)/Sonication	Dec 2014–Oct 2015/77	Suburban (Salento’s peninsula; Italy	Pietrogrande et al. (2018b)
1.23 ± 0.68	-	Methanol:DIW/Sonication	Mar 2014–May 2016/42	Background (Mount Abu; India)	Patel and Rastogi (2018)
2.71–3.42	-	Methanol/Sonication	Apr–Nov 2016/86	Urban and rural (Tehran; Iran)	Rezaei et al. (2018)
1.26–1.35	-	Methanol or DIW/Sonication	Apr–Nov 2016/86	Urban and rural (Tehran; Iran)	Rezaei et al. (2018)
~ 0.07 to ~ 0.15^c^	-	DIW/Vortex mixer	-^b^	Urban site (Nice and Passy; France)	Calas et al. (2017)
0.32–0.80	-	DIW/Sonication	2013–2016/30	Urban (Lecce; Italy)	Chirizzi et al. (2017)
0.04–0.15^a^	-	DIW/Sonication	Jan–Oct 2013/80	Urban (Milan; Italy)	Perrone et al. (2016)
1.7–3.7^d^	41.9–172.5^d,e^	MeOH/Sonication	Mar–Oct 2009/30	Urban (Netherlands)	Janssen et al. 2014
0.5–2.5	0.4–1.9	Gamble + DPPC solution/Vortex mixer	Jan 2018–Mar 2019/199	Urban and background (Barcelona and Montseny; Spain)	Veld et al. (2023)
~ 0.5	~ 0.25	Gamble + DPPC solution/Vortex mixer	Jan 2016–Dec 2020/253	Rural background (France)	Borlaza et al. (2022)
0.8–3.0	0.6–4.1	Gamble + DPPC solution/Vortex mixer	Jan 2018–May 2019/908	Rural and suburban (Payerne, Basel, Zürich, Bern, and Ticino; Switzerland)	Grange et al. (2022)
~ 0.5	~ 0.25	Gamble + DPPC solution/Vortex mixer	Fen 2012–Dec 2020/434	Background (France)	Weber et al. (2021)
~ 1.5 to ~ 5.0^f^	** ~ **0.2 to ~ 4.0^f^	Gamble + DPPC solution/Vortex mixer	Jan 2015–Dec 2016/^−b^	Urban-near refining areas (Orellana, Sucumbíos and Esmeraldas; Ecuador)	Barraza et al. (2020)
~ 1.2 to ~ 3.2^f^	~ 0.5 to ~ 1.5^f^	Gamble + DPPC solution/Vortex mixer	Mar 2012–May 2015/728	Urban (Marnaz, Passy, Chamonix, Grenoble, Talence, Nice, Port de Bouc; France)	Calas et al. (2019)
2.1^f^	7.1^f^	Gamble + DPPC solution/Vortex mixer	Nov 2013–Oct 2014/98	Urban (Chamonix; France)	Calas et al. (2018)
~ 2 to ~ 8	** ~ **1 to ~ 15	Gamble + DPPC solution/Vortex mixer	Nov 2013–Oct 2014/115	Urban (Chamonix Mont Blanc; France)	Weber et al. (2018)
~ 0.06 to ~ 0.13^c^	-	Gamble + DPPC/Vortex mixer	-	Urban site (Nice and Passy; France)	Calas et al. (2017)
~ 0.06 to ~ 0.12^c^	-	Gamble/Vortex mixer	-	Urban site (Nice and Passy; France)	Calas et al. (2017)
~ 0.02 to ~ 0.03^c^	-	Gamble/Vortex mixer	-	Urban site (Nice and Passy; France)	Calas et al. (2017)
**-**	40.8 ± 13.8^ g^	RTLF/-	Nov 2014–Mar 2015/^−b^	Urban (Kraków; Poland)	Styszko et al. (2017)

^a^Total suspended particulate matter (TSP)

^−^^b^Not reported

^c^nmol min^−1^ µg^−1^

^d^Geometric mean

^e^Expressed as nmol s^−1^ m^−3^

^f^Median

^g^Exprerssed as µg m^−3^ (AA depletion over the 90-min incubation period)

*DIW* deionized water, *DPPC* dipalmitoylphosphatidylcholine, *RTLF* synthetic respiratory tract lining fluid

Recently, OP assessment procedures for PM_10_-associated pollutants have been shifting away from the use of deionized water or buffer solutions and exhaustive procedures (such as ultrasound extraction) towards the use of simulated lung fluids (SLFs) such as Gamble solution (GS), artificial lysosomal fluid (ALF), or synthetic respiratory tract lining fluid (RTLF) as the extracting solution, along with vortex agitation at 37 °C for 2 h (Calas et al. 2017, 2018, 2019; Styszko et al. 2017; Weber et al. 2018, 2021; Barraza et al. 2020; Grange et al. 2022; Borlaza et al. 2022; Veld et al. 2023). These procedures are summarized in Table 1. In this context, the knowledge of OP of PM and PM-bound pollutants in SLFs, using standardized approaches such as in vitro physiologically based extraction test (PBET) that simulate the dissolution processes of PM_10_-bound pollutants in the lungs, is necessary for an accurate prediction of the risk to human health.

Due to the lack of OP data in PM_10_ samples using SLF and in vitro standardized approaches, this research aims to assess the OP^DTT^ and OP^AA^ of PM_10_ samples collected at an urban site in the bioaccessible fractions of PM after an in vitro PBET using GS. Although the correlation of OP with the content of water-soluble PM-associated pollutants (including major ions and metal(oid)s (Janssen et al. 2014; Perrone et al. 2016; Pietrogrande et al. 2018b, 2022a, b; Giannossa et al. 2022; Clemente et al. 2023), organic and elemental carbon (Perrone et al. 2016), and PAHs (Janssen et al. 2014; Perrone et al. 2016; Pietrogrande et al. 2022b)) has been extensively assessed, the correlation of OP with bioaccessible fraction of metal(oid)s (Calas et al. 2018, 2019) and PAHs (Calas et al. 2018, 2019; Weber et al. 2018) has been studied or reported in a few PM_10_ samples. This research will also include the measurement of total and bioaccessible concentrations of metal(oid)s and PAHs, as well as the assessment of the correlation between OP^DTT^ and OP^AA^ with major ions, equivalent black carbon (eBC), and UV-absorbing particulate matter (UVPM).

## Materials and methods

### Chemicals and reagents

Chemicals and reagents used in this study are shown in the Supplementary Material Section.

### PM_10_ sample collection

PM_10_ samples were collected during four seasons in 2017 (from January 1st to December 27th, 2017, on weekdays) at an urban site of A Coruña city, an Atlantic coastal city in the northwest of Spain. The sampling site is located 350 m from the A Coruña harbour (coordinates: 43° 21′ 16.0″ N 8° 23′ 22″ W) and is 5 m above sea level. A Coruña city is the main industrial and financial center of the north of Galicia, with almost 250,000 inhabitants. The climate of the site is humid oceanic, with low thermal fluctuation, copious rainfall, and prevailing winds from the northwest. The sources of PM are attributed to traffic and local activities, as well as industrial emissions and biomass burning. Additionally, due to the proximity to the sea, there is a noticeable contribution of marine aerosol (Moreda-Piñeiro et al. 2015).

PM_10_ samples were collected using an automatic high-volume sampler DIGITEL DHA–80 (Hegnau, Switzerland) equipped with a 10-µm-diameter cut-off particle separator onto 15-cm-diameter quartz fibre filters (Ahlstrom Munksjo MK360, Falun, Sweden) at 30 m^3^h^−1^ for 24 h (00:00–23:59, UTC), following European Norm 12,341 (EN 12341:2015) (UNE 2015).

To determine PM_10_ mass concentrations, filters were conditioned at 20 ± 1 °C and a relative humidity of 50 ± 5% for 48 h (UNE 2015) before being weighted using a microbalance (Sartorius Genius, Gottingen, Germany) with a precision of 0.01 mg. To decrease gravimetric bias, several field blanks were collected. After gravimetric determination, filters were kept in aluminium foil, placed inside hermetically seal plastic bags, and stored at –18 °C in a freezer until analysis. Directive 2008/50/EC (EU 2008) was taken into account to establish the lowest time coverage for indicative measurements. Sixty-five samples (one or two samples per week, distributed randomly over the year) were selected for the determination of the OP.

### In vitro inhalation bioaccessibility procedure

**Fig. 1:**
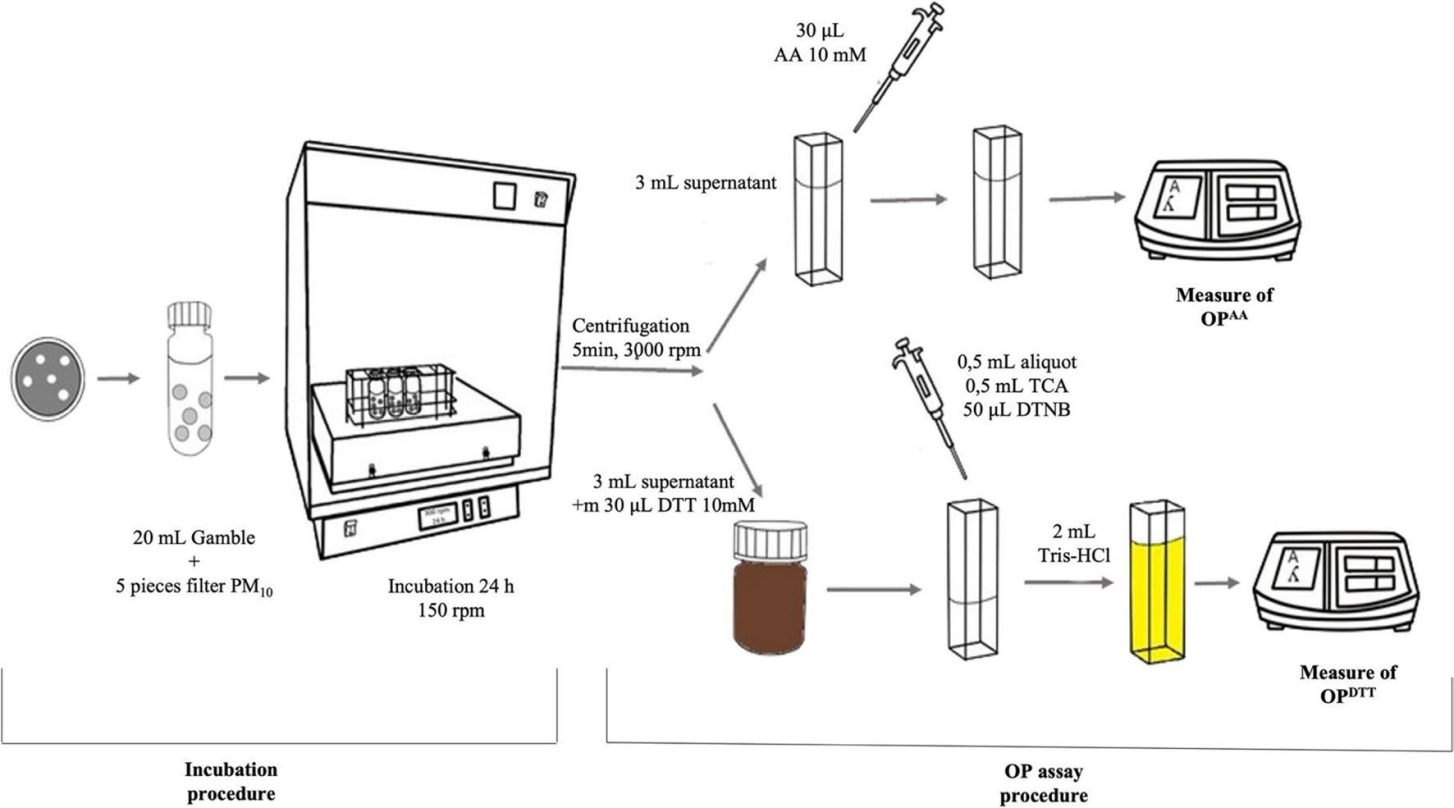
Scheme of the in vitro inhalation bioaccessibility procedure and oxidative potential measuring OP^AA^ and OP^DTT^ assays

Five circular portions of punches with a diameter of 1.2 cm (total filter area of 5.65 cm^2^, PM_10_ mass concentration ranged from 10 to 94 µg m^−3^) were placed in a 50-mL centrifuge tube with 20 mL of GS (pH = 7.4 ± 0.1), resulting in a solid/liquid (S/L) ratio ranging from 1:1000 to 1:125,000 g mL^−1^. The composition of GS is shown in the Supplementary Material Section (Table S1). A S/L ratio higher than 1:1000 was selected, assuming an intake air volume of 20 m^3^ day^−1^ and a total volume lining the lung epithelium of 20 mL (Kastury et al. 2017). The samples were incubated for 24 h at 37 °C and 100 rpm in an incubator shaker (Boxcult incubator and Rotabit orbital-rocking platform shaker, J.P. Selecta, Barcelona, Spain) (Fig. 1) (Kastury et al. 2017). After incubation, the bioaccessible fraction (aqueous phase) was separated from non-bioaccessible fraction by centrifugation (Eppendorf 5804, Madrid, Spain) at 2500 rpm for 10 min. The bioaccessible fraction was then kept at − 20 °C before measurements. Two filter blanks were also obtained for each prepared set of samples.

### Extraction procedure assisted by ultrasound energy

Five circular pieces with a diameter of 1.2 cm (total filter area of 5.65 cm^2^) were extracted using 20 mL of phosphate buffer (0.1 M at pH 7.4) by sonication for 30 min in an ultrasonic bath (J.P. Selecta, Barcelona, Spain) operating at room temperature, a frequency of 37 kHz, and a power of 150 W. After centrifugation, the aqueous soluble fraction was kept at − 20 °C before measurements. Two filter blanks were also obtained for each prepared set of samples.

### PM_10_ oxidative potential assessment

The OP of the collected PM_10_ samples was assessed using the DTT and AA acellular assays following the previous experimental procedures (with few modifications, Fig. 1) (Cho et al. 2005).

#### DTT assay procedure

Thirty microlitres of 10 mM of DTT solution (in 0.4 M phosphate buffer pH = 7.4) was added to 3.0 mL of bioaccessible fraction or phosphate buffer extracts (i.e., time zero). At 0, 5, 10, 20, 30, and 40 min, aliquots of 0.5 mL of the reaction mixture were mixed into a 1.0-cm path length optical quartz cell with 0.5 mL of trichloroacetic acid (TCA) at 10% (v/v) (TCA was added to the mixture at the selected times to end the DTT reaction) and 50 µL of 10 mM DTNB solution in phosphate buffer at pH 7.4 (DTNB was added to react with the residual DTT). After 2 min, 2.0 mL of 0.4 M Tris–HCl buffer (pH 8.9 with 20 mM of EDTA) was added, which leads to the generation of TNB^2−^ (yellow-coloured complex). The concentration of formed TNB^2−^ was measured using a UV–VIS spectrometer (Lambda 6. Perkin Elmer, Norwalk, USA) at 412 nm.

#### AA assay procedure

Three millilitres of the bioaccessible fraction or phosphate buffer extracts was taken into a 1.0-cm path length optical quartz cell, and 30 µL of the 10 mM AA solution was added at zero time. AA depletion (OP^AA^) rates (μM min^−1^) were measured at 265 nm at defined time intervals (after 2.0 min during 30 min).

DTT and AA reduction (OP^DTT^ and OP^AA^, respectively) rates (μM min^−1^) were then determined as the slope of a straight line attained by several data points (absorbance against time) following the procedure described in Pietrogrande et al. (Pietrogrande et al. 2018a). A good linearity (correlation coefficient *R*^2^ > 0.9897 and 0.9980, respectively) was obtained for most of the samples. PM_10_ samples and blank assays were measured three times (RSD less than 14 and 19%, respectively).

The OP^DTT^ and OP^AA^ of the PM_10_ samples were calculated after blank correction by subtracting the mean filter blank activities from the DTT and AA activity. OP^DTT^ and OP^AA^ rates were normalized with the air collected volume (OP^DTT^_V_ and OP^AA^_V_), and the results are expressed in nmol min^−1^ m^−3^. The limits of detection (LODs) were calculated using: *X* + 3 SD criterion (where *X* and SD are the OP^DTT^ or OP^AA^ mean and standard deviations estimated by analyzing 12 procedure blanks) and LOQs (*X* + 10 SD criterion). LOD and LOQ values were 0.001 and 0.003 nmol min^−1^ m^−3^ for OP^DDT^_V_ and 0.05 and 0.07 nmol min^−1^ m^−3^ for OP^AA^_V_.

### Chemical composition of PM_10_: major ions, metal(oid)s, polycyclic aromatic hydrocarbons, and equivalent black carbon and UV-absorbing particulate matter quantification

#### Major ions and trace metal(oid)s quantification

Major ions in PM_10_ samples were quantified, after an aqueous extraction, by zone capillary electrophoresis (ZCE). Metal(oid)s were evaluated, after an acid extraction and in vitro inhalation bioaccessibility procedure, by inductively coupled plasma mass spectrometry (ICP–MS). A brief summary of extraction, quantification, and quality control of major ions in 65 PM_10_ samples and metal(oid)s in PM_10_ samples and inhalation bioaccessible fraction is described in the Supplementary Material Section (Blanco-Heras et al. 2008; Moreda-Piñeiro et al. 2015).

#### PAH extraction and quantification procedures

PAH concentrations in PM_10_ samples, after microwave-assisted extraction, and in the bioaccessible fraction (after pre-concentration by vortex–assisted liquid–liquid micro-extraction (VALLME)) were measured by high-performance liquid chromatography coupled to a fluorescence detector, according to Sánchez–Piñero et al. (Sánchez-Piñero et al. 2021). Detailed procedures and quality control are discussed in the Supplementary Material Section.

#### Equivalent black carbon and UV-absorbing particulate matter quantification

eBC and UVPM were measured by using a Magee Sootscan™ OT–21 (Berkeley, California, USA) transmissometer at 880 nm (a measure of light-absorbing carbon analogous to black carbon) and at 370 nm (a measure of UVPM, an indicator of aromatic organic compounds) (Davy et al. 2017; Greilinger et al. 2019).

### Air mass trajectories

Air mass trajectories were calculated 120 h before the entrance time to the sampling site using the NOAA Hybrid Single-particle Lagrangian Integrated Trajectory Model (HYSPLIT) model (Stein et al. 2015; Rolph et al. 2017). Air mass trajectories data were providing by the Spanish Ministry for the Ecological Transition and the Demographic challenge (MTERD 2023). Air masses were categorized into five groups: class AO represents air masses transported from the Atlantic Ocean (North Atlantic, NA; Northwest Atlantic, NWA; Southwest Atlantic, SWA; West Atlantic, WA), class EU denotes air masses from central and northern Europe, class MED contains air masses transported from the Mediterranean, class NAF refers to air masses with origins in North Africa, and class RE denotes local air masses.

### Data analysis

In order to perform the analytical data treatment, the Kolmogorov–Smirnov test was used for normality assessment of data distribution. Analysis of variance (ANOVA) test was conducted to compare the seasonal means statistically. Spearman rank correlations were employed to identify relationships between different variables. Principal Component Analysis (PCA) was executed using SPSS version 25 (IBM SPSS Statistics, ST, SC., USA). PCA was performed after data set homogenization (half-range and central value transformation), cross-validation, and normalization (Varimax rotation).

## Results and discussion

### Atmospheric particle-bound major ions, metal(oid)s, PAHs, eBC, and UVPM concentrations in PM_10_

The statistical summary (maximum, minimum, mean, and relative standard deviation) for major ions, metal(oid)s, eBC, UVPM, and PAH concentrations in PM_10_ samples during the 1-year sampling and during summer and winter seasons are shown in Table S2-4 (Supporting Information Section). Seasons were determined based on climatological conditions: warm season (April–September) and cold season (October–March). Throughout the entire sampling period, the predominant ion was SO_4_^2−^ (539–15,300 ng m^−3^), followed by Cl^−^ (< 0.15–10,200 ng m^−3^), Na^+^ (157–7330 ng m^−3^), NH_4_^+^ (< 0.17–8590 ng m^−3^), and NO_3_^−^ (196–4960 ng m^−3^). The ions Ca^2+^ (14.5–2630 ng m^−3^) and Mg^2+^ (21.4–1370 ng m^−3^) were present in lower concentrations (Fig. S1, Supporting Information Section). The contributions of major ions to PM_10_ fractions accounted for 61.0 ± 20%, with the oceanic contribution (Cl^−^ plus Na^+^) (20.0 ± 12.4%) being higher than the contribution from other continental or Mediterranean European regions. Similar trends were observed during both the warm and cold seasons for most ions, eBC, and UVPM (Table S2 and Fig. S1, Supporting Information Section). However, ANOVA results indicated statistically significant differences (95.0% confidence level) between the summer and winter seasons (*p*-values of the *F*-test lower than 0.05) for SO_4_^2−^ (*p*-value = 0.005).

The contributions of metal(oid)s to PM_10_ during the entire sampling period accounted for 3.4 ± 3.7%. High levels of Al and Fe (Table S3, Supporting Information Section) were found in PM_10_ samples during the entire sampling period (< 150–6490 and 43.9–3130 ng m^−3^ for Al and Fe, respectively) as well as during the summer (< 150–806 and 43.9–772 ng m^−3^ for Al and Fe, respectively) and winter (< 150–6490 and 73.8–3130 ng m^−3^ for Al and Fe, respectively) seasons. The range of trace metal(oid)s (ng m^−3^) followed the order of Mn > Zn > Pb > Cu > Ni > V > Sr > Cr > Sb > Cd ~ As > Bi > Se during 1-year period and both seasons (Table S3 and Fig. S2, Supporting Information Section). No statistically significant seasonal changes were found after performing the ANOVA test.

The contribution of PAHs to PM_10_ mass accounted for only 0.032 ± 0.030% during the entire sampling period. Benzo(b)fluoranthene (BbF) and benzo(e)pyrene (BeP) were the major PAHs during the 1-year sampling period, with average concentrations of 1.6 and 1.5 ng m^−3^, respectively. This pattern was consistent during both the summer and winter seasons (Table S4 and Fig. S3, Supporting Information Section). Naph, Ace, Fl, and Ant (volatile PAHs existing in the gas phase) were present in very low levels, with concentrations below the limit of quantification (LOQ) in almost all PM_10_ samples. Additionally, concentrations of carcinogenic PAHs (benzo(a)anthracene, BaA; chrysene, Chry; BbF; benzo(k)fluoranthene, BkF; benzo(a)pyrene, BaP; dibenzo(a.h)anthracene, DBahA; and indeno(1.2.3 c.d)pyrene, IP) and non-carcinogenic PAHs (phenanthrene, Phe; fluoranthene, Ft; pyrene, Pyr; BeP; and benzo(g.h.i)perylene, BghiP) did not show significant differences between seasons.

The annual-averaged levels obtained for As, Cd, Ni, and BaP were 0.34 ± 0.30 ng m^−3^, 0.13 ± 0.23 ng m^−3^, 5.8 ± 5.9 ng m^−3^, and 0.48 ± 0.54 ng m^−3^ for As, Cd, Ni, and BaP, respectively. These levels did not surpass the annual target concentrations set by the European Directive in PM_10_ (6.0 ng m^−3^, 5.0 ng m^−3^, 20 ng m^−3^, and 1.0 ng m^−3^ for As, Cd, Ni, and BaP, respectively) (EU 2004).

The low seasonal variation of eBC and UVPM, as well as anthropogenic compounds (NO_3_^−^, NH_4_^+^, Co, Mn, Pb, Zn, and PAHs), observed at this Atlantic site of the northwest of Spain can be attributed to the predominant entrance of clean air masses at the sampling site, mainly originating from the Atlantic Ocean. The major air masses from the Atlantic Ocean accounted for 80% of the days, including the North Atlantic (NA, 12.3%), Northwest Atlantic (NWA, 35.9%), Southwest Atlantic (SWA, 9.6%), and West Atlantic (WA, 21.9%) (Fig. S4, Supporting Information Section).

### In vitro metal(oid)s and PAHs bioaccessible concentrations in PM_10_

Table S3-4 (Supporting Information Section) show the summary data of metal(oid)s and PAH inhalation bioaccessible concentrations (mean, maximum, minimum, and RSD) in PM_10_ samples during the 1-year sampling period and the summer and winter seasons. During the 1-year sampling period, metal(oid) concentrations varied in the range of < 1.4–5.0, < 0.25–2.7, < 0.51–2.1, < 0.8–7.7, < 0.32–4.5, < 0.44–2.8, < 0.44–1.8, and < 2.8–4.7 ng m^−3^ for Al, Cr, Cu, Fe, Mn, Ni, V, and Zn, respectively (Fig. S5, Supporting Information Section). Additionally, As, Bi, Cd, Pb, Sb, Se, and Sr concentrations were below the LOQs (0.88, 0.47, 1.1, 0.28, 0.58, 3.2, and 0.84 ng m^−3^ for As, Bi, Cd, Pb, Sb, Se, and Sr, respectively) in all inhalation bioaccessible fractions. BbF and BeP were the most abundant PAHs observed throughout the year (0.25 and 0.21 ng m^−3^ for BbF and BeP, respectively), as well as during both the summer (0.27 and 0.22 ng m^−3^ for BbF and BeP, respectively) and winter (0.23 and 0.20 ng m^−3^ for BbF and BeP, respectively) seasons (Fig. S6, Supporting Information Section). Finally, inhalation bioaccessible concentrations of PAHs did not present statistically significant seasonal changes between warm and cold seasons. As expected, bioaccessible metal(oid)s and PAHs concentrations were lower than total concentrations in PM_10_ samples, indicating that these compounds were partially solubilized in GS.

**Fig. 2:**
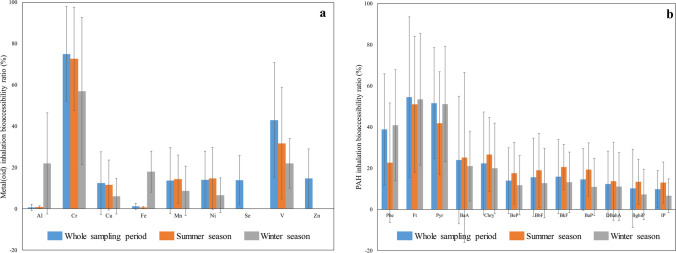
Metal(oid)s (**a**) and PAHs (**b**) in vitro inhalation bioaccessibility ratios (expressed as percentage %) obtained for PM_10_ samples

Figure 2a–b show the metal(oid)s and PAH inhalation bioaccessibility ratios, *B*_acc_, which were calculated using the following equation: *B*_acc_ (%) = (*C*_bioaccessible fraction_/*C*_total_)x100, where *C*_bioaccessible fraction_ and *C*_total_ are the compound concentrations in GS and in PM_10_ samples, respectively. Cr, V, Phe, Ft, and Pyr appear to be highly bioaccessible compounds (mean *B*_acc_ ratios higher than ≈40%), while other metal(oid)s such as Al, Cu and Fe, and PAHs with 6 condensed rings presented low *B*_acc_ ratios (less than ≈10%). The large range of bioaccessible ratios of metal(oid)s and PAHs could indicate the different chemical composition of PM_10_ samples.

### Oxidative potential of PM_10_ samples

**Fig. 3:**
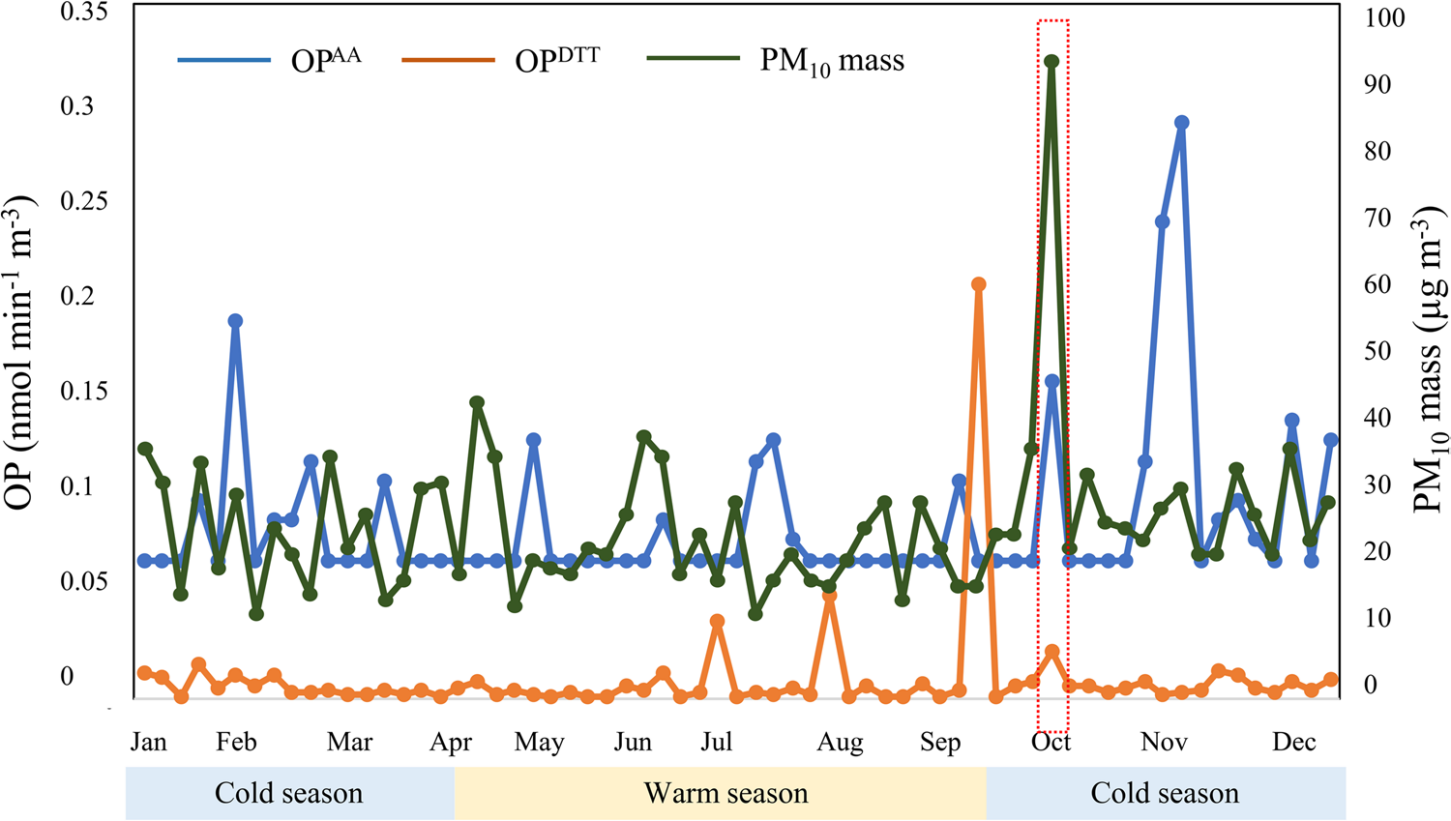
Temporal variation of OP^AA^ and OP^DTT^ (nmol min^−1^ m^−3^) and PM_10_ mass (µg m^−3^) during the study period. The highlighted red square shows days with Saharan dust intrusion

Figure 3 shows the results of PM_10_-induced ROS activity by DTT (OP^DTT^_V_) and AA (OP^AA^_V_) assays in inhalation bioaccessible fractions of 65 PM_10_ samples. The statistical summary for OP^DTT^_V_ and OP^AA^_V_ during the 1-year sampling period and warm and cold seasons is shown in Table 2. OP^DTT^_V_ and OP^AA^_V_ obtained during the 1-year sampling period were in the ranges of < 0.006–0.21 and < 0.07–0.29 nmol min^−1^ m^−3^, respectively, with PM_10_ mass range values between 10 and 42 μg m^−3^ (excluding Saharan dust intrusion episode during October 15th, 2017) (Fig. S7, Supporting Information Section); data were supplied by the Spanish Ministry for the Ecological Transition and the Demographic challenge (MTERD 2020). Additionally, a significant contribution to PM_10_ mass is due to main sea salt ions (Cl^−^ and Na^+^).

**Table 2:** Maximum (Max), minimum (Min), mean, and standard deviation (nmol min^−1^ m^−3^) of oxidative potential in the bioaccessible fraction in PM_10_ samples

	Max	Min	Mean	SD
Whole sampling period				
OP^DTT^	0.21	< 0.006	0.01	0.026
OP^AA^	0.29	< 0.07	0.09	0.041
Summer season				
OP^DTT^	0.21	< 0.006	0.015	0.041
OP^AA^	0.13	< 0.07	0.079	0.019
Winter season				
OP^DTT^	0.024	< 0.006	0.007	0.005
OP^AA^	0.29	< 0.07	0.096	0.049

As expected, OP^DTT^_V_ and OP^AA^_V_ values found were lower than the data reported for PM_10_ samples in other urban sites of Spain and around the world when using deionized water (DIW), methanol, or phosphate buffer (0.1 M at pH 7.4) and vortex/shaking/rotating or sonication (Table 1). The low surface tension of GS and the presence of chelating agents in GS composition could explain the low OP values (Moufarrej et al. 2020; Cigánková et al. 2021). Additionally, the use of ultrasounds enhanced the solubilization of induced ROS activity compounds from PM_10_ samples. The overestimation of OP^DTT^ and OP^AA^ when sonication was used has been confirmed after extracting several PM_10_ samples using phosphate buffer (0.1 M at pH 7.4) (20 mL) as an extracting phase and a sonication (30 min). OP values obtained were twice (0.12 ± 0.12 and 0.28 ± 0.013 nmol min^−1^ m^−3^ for OP^DTT^_V_ and OP^AA^_V_, respectively) than OP values when PBET extraction (0.05 ± 0.008 and 0.12 ± 0.008 nmol min^−1^ m^−3^ for OP^DTT^_V_ and OP^AA^_V_, respectively) was used for selected samples. The high surface tension of phosphate buffer solution and the absence of chelating agents, in contrast with GS (Cigánková et al. 2021), along with ultrasonic waves triggering the pyrolysis of the molecules present inside the cavitation bubbles, resulting in the significant production of free radicals (Massimi et al. 2020), could explain the high OP values obtained when using phosphate buffer solution and ultrasound energy.

In addition, the OP values shown in Table 2 are higher than the OP values reported at several urban, rural, suburban, and background sites in Spain, France, and Switzerland (Table 1) when using GS plus dipalmitoylphosphatidylcholine (DPPC) and vortex as the extraction procedure (Veld et al. 2023; Borlaza et al. 2022; Weber et al. 2021). The filtration avoidance after GS + DPPC and vortex mixing treatment (to contain both water-soluble and insoluble particles for PO assessment) could explain the high OP values reported (Veld et al. 2023; Borlaza et al. 2022; Weber et al. 2021).

Due to the prevalence of clean air masses from Atlantic Ocean (Fig. S4, Supporting Information Section) during the sampling period, OP^DTT^_V_ and OP^AA^_V_ during the winter season were found to be similar to those during the summer season (*p*-values of 0.892 and 0.830 for OP^DTT^_V_ and OP^AA^_V_, respectively).

### Correlations between OP-PM_10_ and PM_10_ sources

A statistical study based on Spearman correlations between OP^DTT^_V_ and OP^AA^_V_ and major and minor constituents of PM_10_ samples (major ions and total metal(oid)s, Σ_12_PAHs, eBC, and UVPM concentrations) was conducted. Previously, the normality of data distribution was assessed by the Kolmogorov–Smirnov test (values of these statistics lower than 0.05 indicate significant departures from normality).

The calculated Spearman correlation coefficients and *p*-values are given in Table 3. Several metal(oid)s such as Cu, Zn, Cr, Fe, Mn, Ni, and V (some of them, main markers of traffic and wear form brake lining and tires and combustion) bound to PM_10_ particles are known to stimulate the hydroxyl radicals generation (Fenton reaction), resulting PM-catalyzed generation of superoxide anion and hydrogen peroxide (Cho et al. 2005; Pant et al. 2015). Additionally, although PAHs are not likely to contribute to OP^DTT^ by direct chemical mechanism, PAHs act as surrogates of redox-active PM sources (Ntziachristos et al. 2007).

**Table 3:** Spearman correlation coefficients and *p*-value (in brackets) between OP^DTT^ and OP^AA^ and total major ions, eBC, UVPM, PM_10_ mas, metal(oid)s, and PAH summations of 12 PAHs (Σ_12_PAHs). Statistical significance represented by ** for *p* < 0.01, and *for *p* < 0.05

	OP^DTT^	OP^AA^
Cl^−^	− 0.127 (− 0.313)	− 0.011 (0.932)
NO_3_^−^	0.062 (0.622)	− 0.201 (0.108)
SO_4_^2−^	− 0.035 (0.785)	− 0.204 (0.103)
NH_4_^+^	0.079 (0.532)	− 0.06 (0.636)
K^+^	0.343** (0.005)	0.061 (0.628)
Na^+^	− 0.026 (0.836)	− 0.054 (0.668)
Ca^2+^	0.069 (0.586)	− 0.111 (0.381)
Mg^2+^	0.052 (0.682)	− 0.113 (0.370)
eBC	0.272* (0.034)	0.162 (0.212)
UVPM	0.124 (0.365)	0.04 (0.773)
PM_10_ mass	0.311* (0.012)	0.081 (0.519)
Al	0.175 (0.163)	− 0.048 (0.703)
As	0.257* (0.039)	0.050 (0.692)
Bi	0.271* (0.029)	0.14 (0.267)
Cd	0.260* (0.036)	− 0.073 (0.564)
Cr	0.146 (0.247)	0.019 (0.881)
Cu	0.290* (0.019)	0.156 (0.214)
Fe	0.212 (0.090)	− 0.045 (0.720)
Mn	0.169 (0.177)	0.064 (0.611)
Ni	0.041 (0.748)	0.200 (0.110)
Pb	0.243 (0.051)	− 0.055 (0.665)
Sb	0.221 (0.076)	0.087 (0.491)
Se	0.009 (0.942)	− 0.138 (0.272)
Sr	0.190 (0.129)	− 0.055 (0.662)
V	− 0.151 (0.230)	− 0.305* (0.014)
Zn	0.105 (0.403)	0.029 (0.816)
Σ_12_PAHs	0.445** (0.000)	0.284* (0.022)

A moderate positive correlation was observed for As, Bi, Cd, and Cu contents in PM_10_ samples and OP^DTT^_V_ (Spearman R 0.257–0.290, *p* < 0.039), suggesting that these metal(oid)s could increase the OP^DTT^_V_. Several authors have reported that the generation of HOOH• and OH• from PM is mainly attributed to the Fe and Cu content of PM, (Charrier and Anastasio 2012). PM_10_ mass (Spearman R 0.311, *p* = 0.012), K^+^ (Spearman R 0.343, p = 0.005), eBC (Spearman R 0.272, *p* = 0.034), and Σ_12_PAHs (Spearman R 0.455, *p* = 0.000) appeared to be positively correlated with OP^DTT^_V_ (Table 3). Additionally, OP^DTT^ was moderately correlated with high-molecular mass-PAHs (including Pyr, BaA, Chry, BeP, BbF, BkF, BaP, DBahA, BghiP, and IP) (Spearman R 0.461–0.354, *p* < 0.004) (Janssen et al. 2014). The positive correlation of eBC and Σ_12_PAHs with OP suggests that organic carbon is an important driver of ROS activity (Styszko et al. 2017). Similar results have been reported for PM_10_ mass and traffic-related PM components (eBC, Cu, and Σ_12_PAHs) (Chao et al. 2005; Janssen et al. 2014; Calas et al. 2017, 2018; Pietrogrande et al. 2018b, 2022a, b), Cd (Perrone et al. 2016), and for K^+^ at several sites in Milan (Italy) (Calas et al. 2018; Pietrogrande et al. 2018b, 2022a, b; Clemente et al. 2023).

No correlation between OP^AA^_V_ measures and PM_10_ mass concentration was obtained, suggesting that OP could be more influenced by PM_10_ composition rather than by PM_10_ mass concentration. Conversely, V (associated with residual oil combustion (Styszko et al. 2017)) concentration was negative correlated with OP^AA^_V_ (Spearman R − 0.305, *p* = 0.014), suggesting that OP^AA^_V_ could be reduced in PM_10_ samples with a high V content. Although it is in contrast to Perrone et al., Barraza et al. and Pietrogrande et al. in which a positive correlation between OP^AA^_V_ and V has been reported (Perrone et al. 2016; Barraza et al. 2020; Pietrogrande et al. 2021), several studies have shown a negative non-significant correlation between OP^AA^_V_ and V (Janssen et al. 2014; Pietrogrande et al. 2018b). Ni showed a significant association with the OP^AA^_V_ response (Spearman R 0.368, *p* = 0.015), in agreement with previous studies (Pietrogrande et al. 2022b). Ni is known to enhance the radical hydroxyl production in the presence of ascorbic acid when it comes into contact with biological cells. Σ_12_PAHs were moderately positively correlated with OP^AA^_V_ (Spearman R 0.284, *p* = 0.022), with generally the highest correlations for Pyr, BaA, Chry, BeP, BbF, BkF, BaP, DBahA, BghiP, and IP (Spearman R 0.259–0.318, *p* =  < 0.037), in agreement with several studies (Janssen et al. 2014; Calas et al. 2018).

Correlations between metal(oid)s and PAHs bioaccessible concentrations with OP were also studied (Table 4). Good positive correlations were observed between Σ_12_PAHs bioaccessible concentration and both OP (Spearman R 0.415, *p* = 0.001 and Spearman R 0.378, *p* = 0.002 for OP^DTT^_V_ and OP^AA^_V_, respectively). Additionally, bioaccessible concentrations of Cu and Ni were observed to be positively correlated with OP^DTT^_V_ (Spearman R 0.345, *p* = 0.008) and OP^AA^_V_ (Spearman R 0.368, *p* = 0.015), respectively. On the other hand, V bioaccessible concentration was negatively correlated with OP^AA^_V_ (Spearman R − 0.305, *p* = 0.014).

As can be seen, major components of PM_10_ (Cl^−^ and Na^+^ (sea spray source) and Ca^2+^ (soil source) did not correlate with OP^DTT^_V_ and OP^AA^_V_. These low correlations are compatible with previous studies (Patel and Rastogi 2018; Weber et al. 2021).

**Table 4:** Spearman correlation coefficients and *p*-value (in brackets) between OP^DTT^ and OP^AA^ and bioaccessible metal(oid)s and PAH summations of 12 PAHs (Σ_12_PAHs) concentrations. Statistical significance represented by ** for *p* < 0.01, and *for *p* < 0.05

	OP^DTT^	OP^AA^
Al	0.067 (0.234)	− 0.127 (0.657)
Cr	− 0.029 (0.930)	− 0.017 (0.718)
Cu	0.345** (0.009)	0.168 (0.210)
Fe	0.212 (0.090)	− 0.045 (0.721)
Mn	0.169 (0.177)	− 0.064 (0.611)
Ni	0.107 (0.494)	0.368* (0.015)
Sb	0.227 (0.071)	0.071 (0.576)
V	− 0.151 (0.230)	− 0.305* (0.014)
Zn	0.170 (0.263)	0.077 (0.615)
Σ_12_PAHs	0.415** (0.001)	0.378** (0.002)

Several moderate to strong correlation between metal(oid)s (total and bioaccessible concentrations) were found (Tables S5-6); the interaction of metal(oid)s could catalyze combined reactions with PM oxidative activity (Shi et al. 2003; Styszko et al. 2017). The discrimination of the data according seasonality, i.e., warm and cold seasons, does not show seasonal trends in the correlation coefficients, signifying a low seasonal variation in the redox-active constituents of PM_10_.

### Principal component analysis

**Fig. 4:**
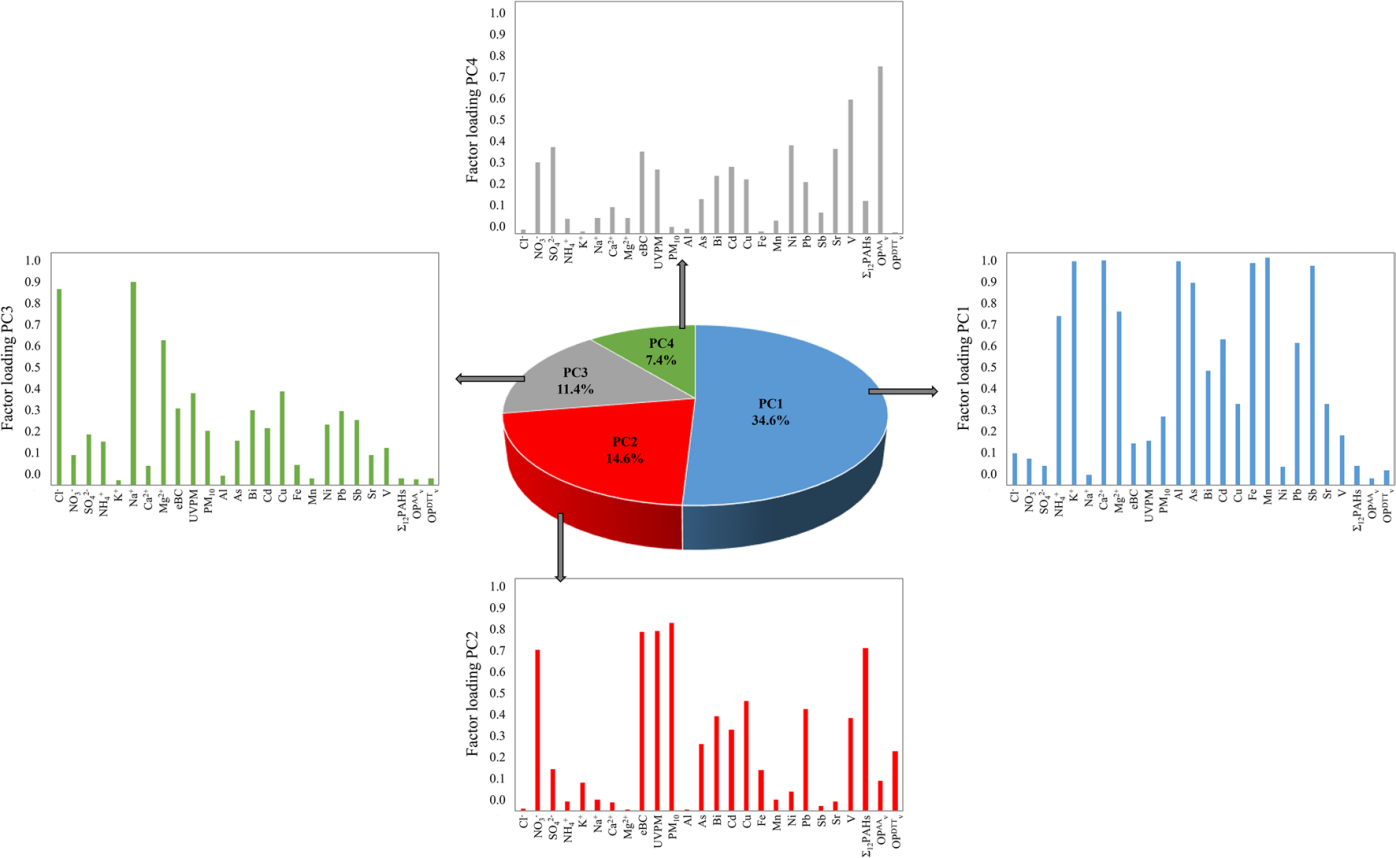
Proportion of variance contributions in percentage from PCA analysis for PM_10_ mass, major ions, metal(oid)s, eBC, UVPM, Ʃ_12_PAHs, OPDTTv, and OP.^AA^_V_ concentrations in PM_10_ samples collected during the 1-year sampling period (*N* = 65)

PCA has been first tried with a data set in which OP^DTT^_V_ and OP^AA^_V_ and PM_10_ mass, eBC, UVPM, major ions, total metal(oid)s, and total Σ_12_PAHs concentrations were the discriminating variables and 65 (1-year sampling period) PM_10_ samples were the objects. Results (Fig. 4) show that 4 principal components (PCs) can explain over 68.0% of the variance. The first factor (PC1), explaining 34.6% of total variance, was associated with crustal/terrestrial (Ca^2+^, Mg^2+^, Al, and Fe) and anthropogenic/biogenic (NH_4_^+^, K^+^, As, Bi, Cd, Mn, Pb, and Sr) sources. Although biogenic species are redox-active, the results show a weak association of these species with OP; in contrast to the result obtained through a univariate approach (K^+^ is positively correlated with OP^DTT^_V_ (Spearman R 0.343, *p* = 0.005)). PC2 (fuel burning and vehicle traffic sources) was loaded with NO_3_^−^, eBC, UVPM, Bi, Cd, Cu, Σ_12_PAHs, and OP^DDT^_V_ (14.6% of the total variance), in agreement with several reported data (Calas et al. 2019). PC3 (sea salt source) offers the highest weights for Cl^−^ and Na^+^ (11.4% of the total variance). These sea salt compounds are not redox-active; thus, they are not associated to OP. Also, PC4 includes OP^AA^_v_, SO_4_^2−^, Ni, and V explaining 7.4% of total variance. The association of traffic emission tracers (Ni and V) and SO_4_^2−^ − with OP^AA^_v_, has also been reported (Strak et al. 2012; Fang et al. 2016).

**Fig. 5:**
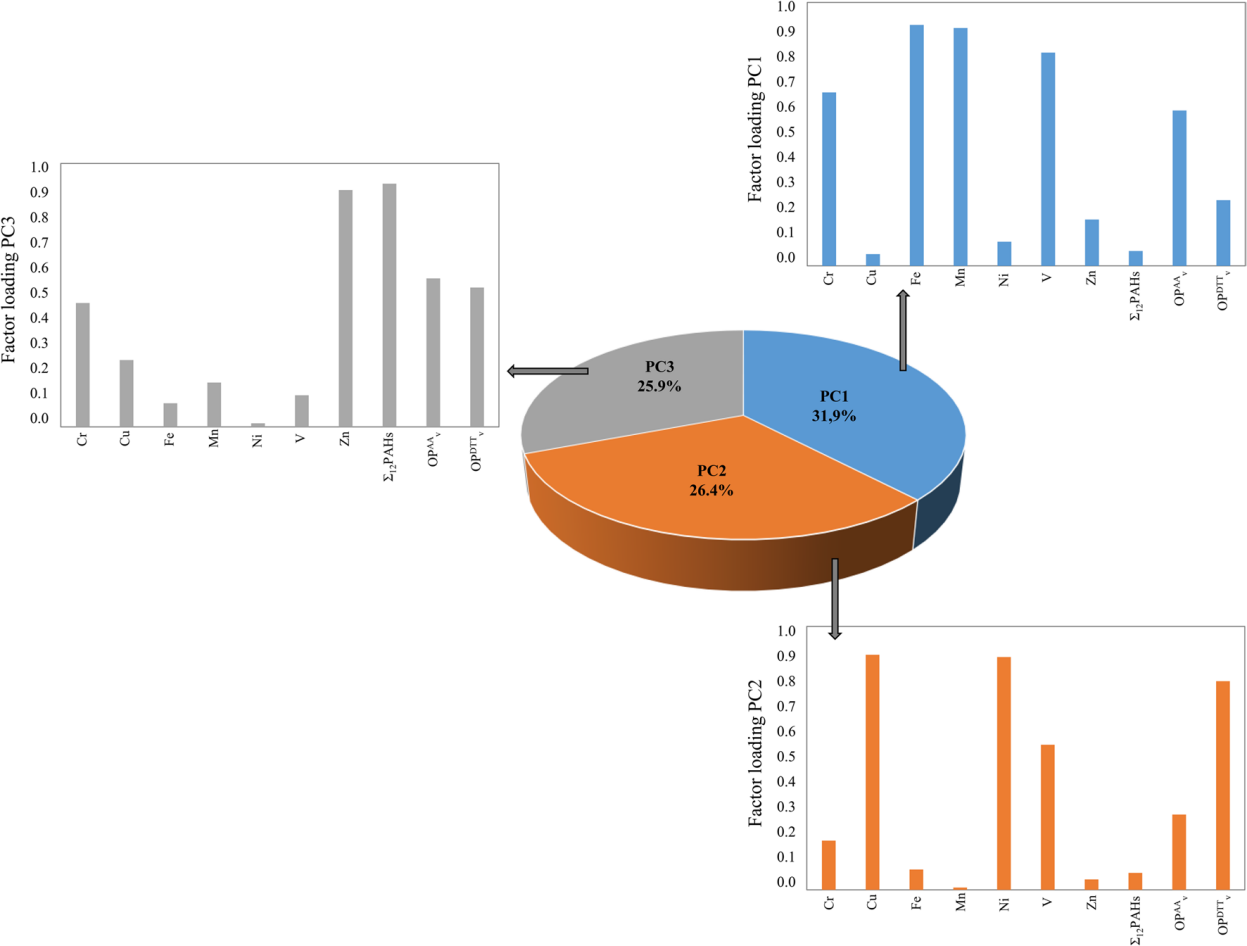
Proportion of variance contributions in percentage from PCA analysis for bioaccessible metal(oid)s and Ʃ_12_PAHs concentrations and OP^DTT^_v_ and OP.^AA^_V_ concentrations in PM_10_ samples collected during the 1-year sampling period (*N* = 65)

PCA has been tried with a dataset in which OP^DTT^_V_, OP^AA^_V_, and bioaccessible metal(oid)s and Σ_12_PAHs concentrations were the discriminating variables, and 65 (1-year sampling period) PM_10_ samples were the objects. The results show that 84.2% of the total variance was explained by 3 PCs (Fig. 5). OP^DTT^_V_ seems to be associated with bioaccessible Cu and Ni concentrations (PC2, 26.4% of the total variance). Additionally, a high weight (0.529) was achieved for OP^DTT^_V_ in the PC3. Factor loadings for OP^AA^_V_ (0.590 and 0.564 for PC1 and PC3, respectively) are very similar in the PC1 (31.9% of the total variance) and the PC3 (25.9% of the total variance), suggesting that Σ_12_PAHs, Cr, Fe, Mn, V, and Zn bioaccessible concentrations are linked with OP^AA^_V_.

The differences observed between both PCA studies using total or bioaccessible concentrations of target compounds may be due to the smaller amount of data associated with the bioaccessible concentrations (see Table S3).

## Conclusions

The oxidative stress of 65 PM_10_ samples was characterized using two procedures (DTT and AA acellular assays) after in vitro PBET methodology using GS (during 24 h at 37 °C) miming inhalation conditions of the human body. OP^DTT^_V_ and OP^AA^_V_ in soluble bioaccessible fraction from PM_10_ collected at a European urban site (Northwest of Spain) means a new contribution to the knowledge in an Atlantic Coastal European region. OP_v_ values obtained at this site were lower than those reported in most other sites in Spain and Europe. This could be due to the low surface tension of GS, the presence of chelating agents in GS composition, and the avoidance of ultrasounds during the extraction process, which may reduce the solubilization of induced ROS activity compounds from PM_10_ samples. The clean Atlantic air masses arriving at the sampling site, which improve the air quality in this region, may also contribute to the reduction in oxidative stress of samples.

In general, no statistically significant seasonal changes were found in PO^DTT^_V_ and PO^AA^_V_ (as well as major ions, metal(oid)s, and PAHs). Data from univariate and multivariate approaches suggest that OP^DTT^_V_ and PO^AA^_V_ are correlated with major ions (K^+^, NO_3_^−^, and SO_4_^2−^) and concentrations of eBC and UVPM. They are also correlated with the total and bioaccessible concentrations of metal(oid)s (such as As, Bi, Cd, Cu, Cr, Fe, Mn, Ni, V, and Zn) and Σ_12_PAHs. These results provide a first step in improving our understanding of the relationship between OP^DTT^_V_ and PO^AA^_V_ and the bioaccessible fraction of PM_10_, as determined by the in vitro PBET methodology. Furthermore, inhalation bioaccessible ratios for Cr, V, Phe, Ft, and Pyr were found to vary from 40 to 70%, indicating that these species might enter the circulation through alveolar absorption. Additionally, in vitro bioaccessible ratios lower than 25% were observed for Al, Cu, Fe, Mn, Ni, Se, Zn, and PAHs with 6 condensed rings.

### Supplementary Information

Below is the link to the electronic supplementary material.

Supplementary file1 (DOCX 1401 kb)

## Data Availability

The datasets generated during and/or analyzed during the current study are not publicly available but are available from the corresponding author on reasonable request.
