# Supplementary material for: Oxidative potential of the inhalation bioaccessible fraction of PM10 and bioaccessible concentrations of polycyclic aromatic hydrocarbons and metal(oid)s in PM10
Source: Environ Sci Pollut Res Int. 2024 Apr 19;31(22):31862–77. doi: 10.1007/s11356-024-33331-9 (PMC11133103; doi:10.1007/s11356-024-33331-9)
Supplement: Supplementary file 1 — Supplementary file1 (DOCX 1401 kb) [file 11356_2024_33331_MOESM1_ESM.docx]

**Supporting Information**

**Oxidative potential of the inhalation bioaccessible fraction of PM_10_ and bioaccessible concentrations of polycyclic aromatic hydrocarbons and metal(oid)s in PM_10_**

Natalia Novo–Quiza, Joel Sánchez–Piñero, Jorge Moreda–Piñeiro, Soledad Muniategui–Lorenzo and Purificación López–Mahía

*University of A Coruña. Grupo Química Analítica Aplicada (QANAP), University Institute of Research in Environmental Studies (IUMA), Department of Chemistry. Faculty of Sciences. Campus de A Coruña, s/n. 15071 – A Coruña. Spain.*

**Corresponding author: Jorge Moreda-Piñeiro.* *Phone number: +34 981 167000. Fax number: +34 981 167065. E–mail address: jorge.moreda@udc.es*

**Abbreviations:**

**Polycyclic aromatic hydrocarbons (PAHs):**

Acenaphthene (Ace); Acenaphtylene (Acy); Acenaphtylene d-8 (Acy-d8); Anthracene (Ant); Anthracene d-10 (Ant-d10); Benzo(a)anthracene (BaA); Benzo(a)pyrene (BaP); Benzo(a)pyrene d-12 (BaP-d12); Benzo(b)fluoranthene (BbF); Benzo(e)pyrene (BeP); Benzo(e)pyrene d-12 (BeP-d12); Benzo(g,h,i)perylene (BghiP); Benzo(g,h,i)perylene d‑12 (BghiP-d12); Benzo(j)fluoranthene (BjF); Benzo(k)fluoranthene (BkF); Chrysene (Chry); Chrysene d-12 (Chry-d12); Dibenzo(a,h)anthracene (DBahA); Dibenzo(a,h)anthracene d‑14 (DBahA-d14); Fluoranthene (Ft); Fluoranthene d-10 (Ft-d10); Fluorene (Fl); Indeno(1,2,3‑c,d)pyrene (IP); Naphthalene (Naph); Naphthalene d-8 (Naph-d8); Phenanthrene (Phe); Phenanthrene d‑10 (Phe-d10); Pyrene (Pyr); Pyrene d-10 (Pyr-d10); Retene (Ret).

**Chemicals**

Ascorbic Acid (Sigma Aldrich, Pharmaceutical Secondary Standard: Certified Reference Material), 1,4-Dithiothreitol (DTT) (Sigma-Aldrich, Germany), 5,5´-Dithiobis(2-nitrobenzoic acid) (DTNB) Sigma-Aldrich, Trizma base NH_2_C(CH_2_OH)_3_ (Primary Standard and Buffer, >99.9%, Sigma-Aldrich, Germany), Sodium di-Hydrogen Phosphate 1-hydrate, for analysis (Panreac, Barcelona, Spain), Ethylenediaminetetracetic Acid Disodium Salt 2-hydrate for analysis- ACS (Panteac, Barcelona, Spain), Trichloroacetic Acid sol. 20% w/v (Panreac, Barcelona, Spain).

Magnesium chloride (MgCl_2_), Sodium sulphate (Na_2_SO_4_), Sodium citrate dihydrate (C_6_H_5_Na_3_O_7_.2H_2_O) and Sodium hydrogen carbonate (NaHCO_3_) were obtained by Sigma-Aldrich (St. Louis, MO, USA). Sodium chloride (NaCl), Potassium chloride (KCl), Calcium chloride dihydrate (CaCl_2_.2H_2_O), Sodium acetate monohydrate (C_2_H_3_O_2_Na. H_2_O) and di-Sodium hydrogen phosphate anhydrous (NaH_2_PO_4_·H_2_O) were purchased from Merck- Millipore (Darmstadt, Germany).

**Major ions extraction and quantification by zone capillary electrophoresis (CE)**

Major inorganic ions were extracted according to the Blanco-Heras et al. procedure (Blanco-Heras et al., 2008). In brief, one quarter of the quartz filters was extracted for15 min by ultrasonication with ultrapure water (2 x 8 mL). At least two procedural blanks were performed for each set of extractions.

Major cations (Na^+^, K^+^, Ca^2+^, NH^4+^ and Mg^2+^) and anions (Cl^−^, NO_3_^−^ and SO_4_^2−^) were analysed by zone capillary electrophoresis (HP3DCE, Agilent, Palo Alto, CA, USA) (Blanco-Heras et al., 2008). Cations were determined using an electrolyte containing 4-aminopyridine 10 mM and 18-crown-6 2.5 mM with pH adjusted to 6.0. The separation of anions was performed using 2.25 mM pyromellitic acid, 0.75 mM hezamethonium and 1.6 mM triethanolamine electrolyte with pH adjusted to 7.7. The detection limits of the method were 9.3 ng m^−3^ for Cl^−^, 15.0 ng m^−3^ for NO_3_^−^, 16.2 ng m^−3^ for SO_4_^2−^, 3.8 ng m^−3^ for NH_4_^+^, 7.6 ng m^−3^ for K^+^, 9.0 ng m^−3^ for Ca^2+^, 4.9 ng m^−3^ for Na^+^, and 3.6 ng m^−3^ for Mg^2+^. K^+^, Ca^2+^, Na^+^ and Mg^2+^ concentrations in SRM 1648a urban particulate matter reference material (National Institute of Standards and Technology, Gaithersburg, MD, USA) and ERM CZ120 fine dust (like PM_10_) (European Commission Joint Research Centre Institute for Reference Materials and Measurements (IRMM), Geel, Belgium) were measured to check the accuracy of the method. After statistical evaluation (by applying a t-test at 95 % confidence level for nine degrees of freedom), good results were achieved.

**Metal(oid)s acid extraction procedure from PM_10_ samples**

Eight circular portions (2.54 cm^2^) of each PM_10_ filters were digested as described in previous papers (Moreda-Piñeiro et al., 2015). In brief, filter portions were transferred to a polytetrafluoroethylene (PTFE) bomb with the addition of 2.5 mL of nitric acid (Baker, Phillipsburg, PA, USA) and 5 mL of hydrofluoric acid (Baker) and heated at 90 °C for 12 h. The mixture was evaporated to dryness after adding 2.5 mL of perchloric acid (Baker) and 1 mL of nitric acid. Once completely dry, 2.5 mL of nitric acid was added, and the solution made up to 25 mL. Two different procedural blanks were performed for each set of acid extractions. All acid extracts were stored at 4 °C in a refrigerator until subjected for trace metal analysis by ICP-MS.

**Metal(oid)s quantification by inductively coupled plasma mass spectrometry (ICP-MS)**

Metal(oid)s in acid extracts of PM_10_ and in bioaccesible fraction (after *in-vitro* inhalation bioaccessibility procedure) were measured by ICP-MS (Thermo Finnigan X Series (Waltham, 125 MA, USA). Detection was performed in the peak jump mode, and monitored ions were m/z 27, 75, 209,111, 53, 65, 56, 55, 60, 208, 121, 78, 88, 51, and 64 for Al, As, Bi, Cd, Cr, Cu, Fe, Mn, Ni, Pb, Sb, Se, Sr, V, and Zn, respectively. [Yttrium](https://www.sciencedirect.com/topics/earth-and-planetary-sciences/yttrium) and [indium](https://www.sciencedirect.com/topics/earth-and-planetary-sciences/indium) (5.0 μg L^−1^), [germanium](https://www.sciencedirect.com/topics/earth-and-planetary-sciences/germanium) (10.0 μgL^−1^), and [scandium](https://www.sciencedirect.com/topics/earth-and-planetary-sciences/scandium) (50.0 μgL^−1^) were selected as internal standards. Optimal ICP-MS conditions have previously published (Moreda-Piñeiro et al., 2015). The limits of quantification for total metal(oid)s quantification were 150 ng m^-3^ for Al, 0.12 ng m^-3^ for As, 0.03 ng m^-3^ for Bi, 0.13 ng m^-3^ for Cd, 2.8 ng m^-3^ for Cr, 1.3 ng m^-3^ for Cu, 20.0 ng m^-3^ for Fe, 0.80 ng m^-3^ for Mn, 1.8 ng m^-3^ for Ni, 0.04 ng m^-3^ for Pb, 0.25 ng m^-3^ for Sb, 0.15 ng m^-3^ for Se, 0.15 ng m^-3^ for Sr, 0.10 ng m^-3^ for V and 9.3 ng m^-3^ for Zn; and 1.4 ng m^-3^ for Al, 0.88 ng m^-3^ for As, 0.47 ng m^-3^ for Bi, 1.1 ng m^-3^ for Cd, 0.25 ng m^-3^ for Cr, 0.51 ng m^-3^ for Cu, 0.80 ng m^-3^ for Fe, 0.32 ng m^-3^ for Mn, 0.44 ng m^-3^ for Ni, 0.28 ng m^-3^ for Pb, 0.58 ng m^-3^ for Sb, 3.2 ng m^-3^ for Se, 0.84 ng m^-3^ for Sr and 0.44 ng m^-3^ for V and Zn (respectively) for metal(loid)s in bioaccessible fractions.The reproducibility and accuracy of the method were assessed by analyzing SRM 1648a urban particulate matter and ERM CZ120 fine dust (like PM_10_). The results of reproducibility of the method were lower than 18% for all metal(oid)s. Concentrations found in SRM 1648a and ERM CZ120 were in good agreement with the certified values after statistical evaluation by applying a t-test at a 95% confidence level for seven degrees of freedom.

**PAHs extraction and clean-up from PM_10_ samples**

Four circular pieces (4.52 cm^2^) of PM_10_ were extracted by using a 1:1 mixture of hexane and acetone (15 mL) and assisted by a microwave energy (ETHOS SEL microwave system, Milestone, Sorisole, BG, Italy), as described in previous papers ([Piñeiro-Iglesias et al., 2003](https://www.sciencedirect.com/science/article/pii/S0045653520320427#bib59), [2004](https://www.sciencedirect.com/science/article/pii/S0045653520320427#bib58)). After cooling to room temperature, the extracts were filtered (MN GF-6 0.6 mm, Macherey-Nagel) and evaporated in rotary evaporator to approximately 1 mL (bath temperature of 35 °C and 475 mbar of pressure). The extracts were cleaned-up using Supelclean™ LC-Si SPE Tubes (Supelco, Steinheim, Germany), pre-cleaned with 6 mL of hexane. The elution was performed using 6.5 mL of hexane, followed by 10 mL of dichloromethane: hexane (30:70) through each clean-up column. All eluates were collected and concentrated using a TurboVap® II Concentration Workstation (Biotage, Uppsala, Sweden) to 500 μL and then to dryness by a stream of N_2_. The residue was dissolved in 500 μL of acetonitrile for the analysis.

**PAHs preconcentration and clean-up from bioaccessible fractions of PM_10_ samples**

The bioaccessible fraction was pre-concentrated and cleaned-up using vortex-assisted liquid-liquid microextraction (VALLME) according to Sánchez-Piñero et al. (Sánchez-Piñero et al., 2021). In brief, 15 μL of surrogate standards (anthracene-d10, Ant-d10 and benzo(e)pyrene-d12, BeP-d12) (Dr. Ehrenstorfer GmbH, Augsburg, Germany) (0.5 μg mL^−1^ in acetonitrile) were added to a bioaccessible fraction and extracted with 300 μL of hexane (Merck-Millipore). The mixture was agitated on a Vibrax IKA VXR basic agitator (Staufen, Germany) at 1500 rpm for 5 min. Then, centrifugation was carried out for 5 min at 3500 rpm (Eppendorf 5804, Madrid, Spain), the organic fraction was collected and concentrated by gentle N_2_ stream and reconstituted with 300 μL of acetonitrile (Merck-Millipore), and finally filtrated through 0.45 μm PTFE syringe filter (Phenomenex, Torrance, CA, USA).

**PAHs quantification by high-performance liquid chromatography (HPLC)**

PAHs were measured by using a high-performance liquid chromatograph system (Waters, Milford, MA, USA) equipped with programmable fluorescence detector (Waters 2475). A Waters^®^ PAH C_18_ (250×4.6 mm i.d., 5 µm) column was used for separation. Based on the work by Maria Amado et al (Fernández-Amado et al., 2016), a chromatographic separation with gradient elution using acetonitrile and water as mobile phases was employed.

External calibration was used for quantifying PAHs in PM_10_, while relative response factors (RRF) with respect to labelled surrogate standards were used for quantifying bioaccessible PAHs. The limits of quantification (LOQs) for *in*−*vitro* inhalation bioaccessibility−VALLME−HPLC-FLD and total PAHs extraction (MAE−HPLC−FLD) procedures ranged from 2.9 pg m^−3^ (anthracene) – 68.0 pg m^−3^ (Pyrene) for *in vitro* bioaccessibility−VALLME−HPLC-FLD, and from 5.3 pg m^−3^ (anthracene) to 561 pg m^−3^ (naphthalene) for MAE−HPLC-FLD. The inter-day precision and accuracy of the analytical procedure were estimated by analysing the PM_10_-like reference material for organics ERM-CZ100 (European Commission Joint Research Centre Institute for Reference Materials and Measurements (IRMM), Geel, Belgium). All PAHs demonstrated good inter-day precision, with RSDs ranging from 6.0% to 16.2%. The accuracy of the MAE−HPLC−FLD method was assessed for 12 PAHs with certified values available in ERM-CZ100. After statistical evaluation by applying a t-test at 95% confidence level for five degrees of freedom, *t_cal_* values obtained for all certificated PAHs are lower than *t_tab_* value of 2.02 (t-Student for 5 degrees of freedom at a confidence level of 95%). Successful validation results of the *in*−*vitro* bioaccessibility VALLME−HPLC-FLD method, as demonstrated by a mass balance study, were achieved (Sánchez-Piñero et al., 2021).

**Table S1.** Composition (g L^-1^) of Gamble’s solution used as simulated lung fluid for inhalation bioaccessibility assessment (Colombo et al., 2008; Midander et al., 2007).

| Reagent | Formula | Concentration |
| --- | --- | --- |
| Magnesium chloride^a^ | MgCl_2_ | 0.095 |
| Sodium chloride^b^ | NaCl | 6.019 |
| Potassium chloride^b^ | KCl | 0.298 |
| Disodium hydrogen phosphate^b^ | Na_2_HPO_4_ | 0.126 |
| Sodium sulphate^a^ | Na_2_SO_4_ | 0.063 |
| Calcium chloride dihydrate^b^ | CaCl_2_.2H_2_O | 0.368 |
| Sodium acetate monohydrate^b^ | C_2_H_3_O_2_Na. H_2_O | 0.701 |
| Sodium hydrogen carbonate^a^ | NaHCO_3_ | 2.604 |
| Sodium citrate dihydrate^a^ | C_6_H_5_Na_3_O_7_.2H_2_O | 0.097 |
| Supplier: ^a^Sigma-Aldrich, St. Louis, MO, USA; ^b^Merck-Millipore Darmstadt, Germany | | |

**Table S2.** Maximum (Max), minimum (Min), and average concentrations of major ions, eBC, and UVPM (expressed as ng m^-3^), PM_10_ mass concentrations (expressed as μg m^-3^), and relative standard deviation (%).

|  | Max | Min | Average | RSD (%) |
| --- | --- | --- | --- | --- |
| *Whole period (N=65)* | | | | |
| Cl^-^ | 10200 | <9.3 | 2230 | 102 |
| NO_3_^-^ | 4960 | 196 | 1600 | 65 |
| SO_4_^2-^ | 15300 | 539 | 2570 | 97 |
| NH_4_^+^ | 8590 | <3.8 | 1240 | 115 |
| K^+^ | 2910 | 74.9 | 318 | 114 |
| Na^+^ | 7330 | 157 | 2150 | 147 |
| Ca^2+^ | 2630 | 14.5 | 234 | 67 |
| Mg^2+^ | 1370 | 21.4 | 274 | 138 |
| eBC | 2404^a^ | 148^a^ | 910^a^ | 59^a^ |
| UVPM | 2558^b^ | 315^b^ | 1265^b^ | 42^b^ |
| PM_10_ mass | 94 | 10 | 23.5 | 50 |
| *Summer season (N=32)* | | | | |
| Cl^-^ | 7674 | 36.4 | 2190 | 87 |
| NO_3_^-^ | 4960 | 363 | 1612 | 71 |
| SO_4_^2-^ | 15300 | 539 | 3453 | 93 |
| NH_4_^+^ | 3630 | 24.3 | 1051 | 91 |
| K^+^ | 965 | 97.8 | 246 | 68 |
| Na^+^ | 5090 | 647 | 2177 | 52 |
| Ca^2+^ | 494 | 44.8 | 198 | 54 |
| Mg^2+^ | 546 | 79.1 | 264 | 48 |
| eBC | 2354^c^ | 148^c^ | 706^c^ | 60^c^ |
| UVPM | 2092^d^ | 315^d^ | 1064^d^ | 10^d^ |
| PM_10_ mass | 42 | 10 | 21 | 38 |
| *Winter season (N=33)* | | | | |
| Cl^-^ | 10200 | <9.3 | 2261 | 116 |
| NO_3_^-^ | 4354 | 196 | 1597 | 60 |
| SO_4_^2-^ | 5360 | 581 | 1723 | 60 |
| NH_4_^+^ | 8590 | <3.8 | 1430 | 124 |
| K^+^ | 2910 | 74.9 | 388 | 124 |
| Na^+^ | 7330 | 157 | 2129 | 81 |
| Ca^2+^ | 2630 | 14.5 | 267 | 164 |
| Mg^2+^ | 1370 | 21.4 | 284 | 93 |
| eBC | 2404^e^ | 263^e^ | 1098^e^ | 51^e^ |
| UVPM | 2558^f^ | 538^f^ | 1477^f^ | 38^f^ |
| PM_10_ mass | 94 | 10 | 26 | 55 |
| ^a^N=61, concentrations of 4 samples exceeded the maximum value for the measurement of the attenuation at 880 nm (filter too dark)  ^b^N=55, concentrations of 10 samples exceeded the maximum value for the measurement of the attenuation at 370 nm (filter too dark)  ^c^N=29, concentrations of 3 samples exceeded the maximum value for the measurement of the attenuation at 880 nm (filter too dark)  ^d^N=29, the concentration of 4 samples exceeds the maximum value for the measurement of the attenuation at 370 nm (filter too dark)  ^e^N=32, the concentration of one sample exceeds the maximum value for the measurement of the attenuation at 880 nm (filter too dark)  ^f^N=27, the concentration of 6 samples exceeds the maximum value for the measurement of the attenuation at 370 nm (filter too dark) | | | | |

**Table S3.** Maximum (Max), minimum (Min), average concentrations (expressed as ng m^-3^), and relative standard deviation (RSD, %) of metal(oid)s bioaccessible and total concentrations found in PM_10_ samples.

|  | Total content | | | | Bioaccessible concentration | | | |
| --- | --- | --- | --- | --- | --- | --- | --- | --- |
|  | Max | Min | Average | RSD | Max | Min | Average | RSD |
| *Whole season (N=65)* | | | | | | | | |
| Al | 6490 | <150 | 383 | 211 | 5.0 | <1.4 | 2.7 | 47 |
| As | 1.9 | <0.12 | 0.34 | 88 | <0.88 | <0.88 | <0.88 | ^-^ |
| Bi | 0.70 | <0.03 | 0.14 | 102 | <0.47 | <0.47 | <0.47 | ^-^ |
| Cd | 1.9 | <0.13 | 0.13 | 179 | <1.1 | <1.1 | <1.1 | ^-^ |
| Cr | 13.2 | <2.8 | 2.4 | 103 | 2.7 | <0.25 | 1.8 | 47 |
| Cu | 27.7 | <1.3 | 6.9 | 89 | 2.1 | <0.51 | 0.86 | 83 |
| Fe | 3130 | 43.9 | 369 | 111 | 7.7 | <0.80 | 4.3 | 49 |
| Mn | 97.3 | <0.80 | 8.1 | 149 | 4.5 | <0.32 | 1.1 | 91 |
| Ni | 24.1 | <1.8 | 5.8 | 101 | 2.8 | <0.44 | 0.81 | 90 |
| Pb | 55.9 | 0.38 | 4.7 | 147 | <0.28 | <0.28 | <0.28 | ^-^ |
| Sb | 9.4 | <0.25 | 2.2 | 83 | <0.58 | <0.58 | <0.58 | ^-^ |
| Se | 0.56 | <0.15 | 0.12 | 98 | <3.2 | <3.2 | <3.2 | 139 |
| Sr | 16.2 | 0.35 | 2.1 | 92 | <0.84 | <0.84 | <0.84 | ^-^ |
| V | 23.4 | 0.66 | 6.3 | 75 | 18 | <0.44 | 2.7 | 116 |
| Zn | 79.9 | <9.3 | 19.7 | 87 | 4.7 | <2.8 | 2.9 | 46 |
| *Summer season (N=32)* | | | | | | | | |
| Al | 806 | <150 | 247 | 89 | 5.0 | <1.4 | 2.2 | 40 |
| As | 1.9 | <0.12 | 0.32 | 101 | <0.88 | <0.88 | <0.88 | ^-^ |
| Bi | 0.70 | <0.03 | 0.12 | 122 | <0.47 | <0.47 | <0.47 | ^-^ |
| Cd | 1.9 | <0.13 | 0.15 | 222 | <1.1 | <1.1 | <1.1 | ^-^ |
| Cr | 13.2 | <2.8 | 2.3 | 131 | 2.7 | <0.25 | 1.7 | 70 |
| Cu | 27.7 | <1.3 | 5.2 | 113 | 1.3 | <0.51 | 0.6 | 87 |
| Fe | 772 | 43.9 | 267 | 69 | 2.9 | <0.80 | 1.8 | 56 |
| Mn | 24.9 | <0.80 | 6.1 | 78 | 2.4 | <0.32 | 0.87 | 71 |
| Ni | 10.9 | <1.8 | 4.4 | 68 | 2.8 | <0.44 | 0.65 | 75 |
| Pb | 55.9 | 0.38 | 4.7 | 204 | <0.28 | <0.28 | <0.28 | ^-^ |
| Sb | 9.4 | <0.25 | 1.7 | 106 | <0.58 | <0.58 | <0.58 | ^-^ |
| Se | 0.46 | <0.15 | 0.14 | 87 | <3.2 | <3.2 | <3.2 | ^-^ |
| Sr | 2.9 | 0.91 | 1.8 | 35 | <0.84 | <0.84 | <0.84 | ^-^ |
| V | 23.4 | 1.2 | 6.9 | 73 | 18 | <0.44 | 2.2 | 139 |
| Zn | 79.9 | <9.3 | 20.9 | 91 | <2.8 | <2.8 | <2.8 | ^-^ |
| *Winter season (N=33)* | | | | | | | | |
| Al | 6490 | <150 | 498 | 223 | 3.7 | <1.4 | 2.0 | 58 |
| As | 1.6 | <0.12 | 0.35 | 81 | <0.88 | <0.88 | <0.88 | ^-^ |
| Bi | 0.47 | <0.03 | 0.17 | 88 | <0.47 | <0.47 | <0.47 | ^-^ |
| Cd | 0.34 | <0.13 | 0.11 | 74 | <1.1 | <1.1 | <1.1 | ^-^ |
| Cr | 7.9 | <2.8 | 2.0 | 76 | <0.25 | <0.25 | <0.25 | ^-^ |
| Cu | 21.7 | <1.3 | 8.2 | 75 | 2.1 | <0.51 | 1.1 | 87 |
| Fe | 3130 | 73.8 | 447 | 120 | 7.7 | <0.80 | 4.9 | 57 |
| Mn | 97.3 | 1.2 | 9.7 | 170 | 4.5 | <0.32 | 1.7 | 69 |
| Ni | 24.1 | <1.8 | 6.9 | 109 | <0.44 | <0.44 | <0.44 | ^-^ |
| Pb | 12.6 | 0.61 | 4.7 | 64 | <0.28 | <0.28 | <0.28 | ^-^ |
| Sb | 6.7 | 0.42 | 2.6 | 70 | <0.58 | <0.58 | <0.58 | ^-^ |
| Se | 0.56 | <0.15 | 0.12 | 112 | <3.2 | <3.2 | <3.2 | ^-^ |
| Sr | 16.2 | 0.35 | 2.21 | 118 | <0.84 | <0.84 | <0.84 | ^-^ |
| V | 16.7 | 0.66 | 5.2 | 79 | 3.7 | <0.44 | 1.7 | 68 |
| Zn | 67.8 | <9.3 | 19.9 | 78 | 4.7 | <2.8 | 3.5 | 47 |
| ^a^Not calculated. Values are lower than the LOQ. | | | | | | | | |

**Table S4.** Maximum (Max), minimum (Min), average concentrations (ng m^-3^), and relative standard deviation (RSD, %) of PAHs (individual PAHs and PAH summations of 12 PAHs (Σ_12_PAHs), carcinogenic PAHs (ΣPAH_c_), and non-carcinogenic PAHs (ΣPAH_nc_)) bioaccessible and total concentrations found in PM_10_ samples.

|  | Total content | | | | Bioaccessible concentration | | | |
| --- | --- | --- | --- | --- | --- | --- | --- | --- |
|  | Max | Min | Average | RSD | Max | Min | Average | RSD |
| *Whole season (N=65)* | | | | | | | | |
| Phe | 1.8 | <0.099 | 0.18 | 161 | 0.53 | <0.045 | 0.07 | 117 |
| Ft | 1.5 | <0.057 | 0.33 | 113 | 0.76 | <0.056 | 0.18 | 92 |
| Pyr | 1.3 | <0.083 | 0.31 | 144 | 0.60 | <0.068 | 0.16 | 91 |
| BaA | 1.5 | <0.041 | 0.25 | 128 | 0.30 | <0.003 | 0.06 | 115 |
| Chry | 4.2 | <0.15 | 0.85 | 114 | 1.3 | <0.015 | 0.19 | 112 |
| BeP | 7.5 | <0.18 | 1.5 | 112 | 1.9 | <0.003 | 0.21 | 152 |
| BbF | 8.7 | <0.20 | 1.6 | 111 | 2.1 | <0.013 | 0.25 | 152 |
| BkF | 2.1 | <0.063 | 0.44 | 109 | 0.55 | <0.004 | 0.07 | 142 |
| BaP | 2.2 | <0.046 | 0.48 | 113 | 0.51 | <0.004 | 0.07 | 137 |
| DBahA | 0.54 | <0.022 | 0.081 | 129 | 0.11 | <0.004 | 0.01 | 174 |
| BghiP | 3.9 | <0.10 | 0.88 | 100 | 0.78 | <0.007 | 0.09 | 269 |
| IP | 2.7 | <0.073 | 0.61 | 101 | 0.54 | <0.008 | 0.06 | 158 |
| Σ_12_PAHs | 33.4 | 0.58 | 7.7 | 96 | 9.6 | 3.08 | 1.4 | 118 |
| ΣPAH_c_ | 19.7 | 0.30 | 4.4 | 108 | 5.3 | ^-a^ | 0.69 | 130 |
| ΣPAH_nc_ | 13.7 | 0.28 | 3.3 | 100 | 4.2 | ^-a^ | 0.68 | 109 |
| Summer season (N=32) | | | | | | | | |
| Phe | 1.2 | <0.099 | 0.13 | 189 | 0.19 | <0.045 | 0.03 | 196 |
| Ft | 1.4 | <0.057 | 0.24 | 152 | 0.76 | <0.056 | 0.12 | 134 |
| Pyr | 1.2 | <0.083 | 0.19 | 157 | 0.60 | <0.068 | 0.08 | 149 |
| BaA | 0.70 | <0.041 | 0.12 | 157 | 0.23 | <0.003 | 0.03 | 162 |
| Chry | 4.2 | <0.15 | 0.60 | 160 | 1.3 | <0.015 | 0.16 | 164 |
| BeP | 7.5 | <0.18 | 1.3 | 154 | 1.9 | <0.003 | 0.22 | 190 |
| BbF | 8.7 | <0.20 | 1.4 | 151 | 2.1 | <0.013 | 0.27 | 183 |
| BkF | 2.1 | <0.063 | 0.34 | 158 | 0.55 | <0.004 | 0.07 | 187 |
| BaP | 1.9 | <0.046 | 0.31 | 160 | 0.51 | <0.004 | 0.06 | 187 |
| DBahA | 0.54 | <0.022 | 0.073 | 175 | 0.11 | <0.004 | 0.01 | 231 |
| BghiP | 3.9 | <0.10 | 0.67 | 145 | 0.78 | <0.007 | 0.09 | 191 |
| IP | 2.7 | <0.073 | 0.46 | 149 | 0.54 | <0.008 | 0.06 | 199 |
| Σ_12_PAHs | 33.4 | 0.58 | 5.9 | 146 | 9.6 | 3.08 | 1.2 | 171 |
| ΣPAH_c_ | 19.7 | 0.30 | 3.3 | 153 | 5.3 | ^-a^ | 0.66 | 178 |
| ΣPAH_nc_ | 13.7 | 0.28 | 2.5 | 140 | 4.2 | ^-a^ | 0.54 | 163 |
| Winter season (N=33) | | | | | | | | |
| Phe | 1.8 | <0.099 | 0.22 | 143 | 0.53 | <0.045 | 0.09 | 124 |
| Ft | 1.5 | <0.057 | 0.43 | 88 | 0.68 | <0.056 | 0.23 | 77 |
| Pyr | 1.3 | <0.083 | 0.43 | 134 | 0.58 | <0.068 | 0.22 | 74 |
| BaA | 1.5 | <0.041 | 0.38 | 99 | 0.30 | <0.003 | 0.08 | 86 |
| Chry | 3.8 | <0.15 | 1.1 | 87 | 0.50 | <0.015 | 0.22 | 67 |
| BeP | 5.8 | <0.18 | 1.7 | 81 | 0.85 | <0.003 | 0.20 | 92 |
| BbF | 5.5 | <0.20 | 1.8 | 80 | 1.3 | <0.013 | 0.23 | 105 |
| BkF | 1.5 | <0.063 | 0.53 | 77 | 0.22 | <0.004 | 0.07 | 80 |
| BaP | 2.2 | <0.046 | 0.64 | 87 | 0.23 | <0.004 | 0.07 | 93 |
| DBahA | 0.28 | <0.022 | 0.088 | 90 | 0.04 | <0.004 | 0.009 | 118 |
| BghiP | 1.8 | <0.10 | 1.1 | 70 | 0.28 | <0.007 | 0.08 | 101 |
| IP | 2.7 | <0.073 | 0.75 | 70 | 0.18 | <0.008 | 0.05 | 106 |
| Σ_12_PAHs | 16.2 | 0.68 | 9.1 | 75 | 3.7 | 0.08 | 1.5 | 69 |
| ΣPAH_c_ | 11.2 | 0.33 | 5.3 | 79 | 2.3 | ^-a^ | 0.74 | 77 |
| ΣPAH_nc_ | 13.7 | 0.35 | 3.8 | 74 | 1.9 | 0.03 | 0.82 | 68 |
| ^a^Not calculated, PAHs were lower than the LOQs | | | | | | | | |

**Table S5.** Spearman correlation coefficients and p-value (in brackets) between total metal(oid)s concentrations. Statistical significance represented by *** for p<0.001, ** for p<0.01, and *for p<0.05.

|  | **Al** | **As** | **Bi** | **Cd** | **Cr** | **Cu** | **Fe** | **Mn** | **Ni** | **Pb** | **Sb** | **Se** | **Sr** | **V** | **Zn** |
| --- | --- | --- | --- | --- | --- | --- | --- | --- | --- | --- | --- | --- | --- | --- | --- |
| **Al** |  | 0.631** (0.000) | 0.417** (0.001) | 0.392** (0.001) | 0.393** (0.001) | 0.294* (0.018) | 0.582** (0.000) | 0.641** (0.000) | 0.256* (0.039) | 0.446** (0.000) | 0.421** (0.000) | 0.187 (0.136) | 0.480** (0.000) | 0.439** (0.000) | 0.294** (0.018) |
| **As** |  |  | 0.778** (0.000) | 0.616** (0.000) | 0.520** (0.001) | 0.599** (0.018) | 0.731** (0.000) | 0.687** (0.000) | 0.258* (0.038) | 0.665** (0.000) | 0.750** (0.000) | 0.051 (0.689 | 0.347** (0.005) | 0.387** (0.001) | 0.459** (0.000) |
| **Bi** |  |  |  | 0.676** (0.000) | 0.412** (0.001) | 0.536** (0.018) | 0.594** (0.000) | 0.548** (0.000) | 0.278* (0.025) | 0.630** (0.000) | 0.720** (0.000) | 0.093  (0.460) | 0.320** (0.009) | 0.186 (0.138) | 0.453** (0.000) |
| **Cd** |  |  |  |  | 0.527** (0.000) | 0.392** (0.001) | 0.380** (0.002) | 0.441** (0.000) | -0.007 (0.955) | 0.611** (0.000) | 0.512** (0.000) | 0.034  (0.791) | 0.346** (0.005) | 0.254* (0.041) | 0.579** (0.000) |
| **Cr** |  |  |  |  |  | 0.400** (0.001) | 0.480** (0.000) | 0.474** (0.000) | -0.038 (0.763) | 0.224 (0.073) | 0.402** (0.001) | -0.082  (0.514) | 0.326** (0.008) | 0.106 (0.402) | 0.362** (0.003) |
| **Cu** |  |  |  |  |  |  | 0.665** (0.000) | 0.520** (0.000) | 0.139 (0.271) | 0.424** (0.000) | 0.743** (0.000) | -0.213  (0.089) | 0.111 (0.380) | 0.042  (0.738) | 0.208 (0.097) |
| **Fe** |  |  |  |  |  |  |  | 0.876** (0.000) | 0.144 (0.254) | 0.572** (0.000) | 0.819** (0.000) | 0.097  (0.444) | 0.278* (0.025) | 0.350**  (0.004) | 0.226 (0.070) |
| **Mn** |  |  |  |  |  |  |  |  | 0.202 (0.107) | 0.646** (0.000) | 0.729** (0.000) | 0.161  (0.200) | 0.324** (0.008) | 0.525**  (0.004) | 0.388** (0.001) |
| **Ni** |  |  |  |  |  |  |  |  |  | 0.158 (0.210) | 0.127 (0.315) | 0.054  (0.671) | 0.140 (0.267) | 0.195  (0.119) | -0.011 (0.933) |
| **Pb** |  |  |  |  |  |  |  |  |  |  | 0.638** (0.000) | 0.089  (0.483) | 0.187 (0.135) | 0.459**  (0.000) | 0.559** (0.000) |
| **Sb** |  |  |  |  |  |  |  |  |  |  |  | -0.003  (0.984) | 0.213 (0.089) | 0.302*  (0.015) | 0.283* (0.023) |
| **Se** |  |  |  |  |  |  |  |  |  |  |  |  | 0.063 (0.616) | 0.320**  (0.003) | 0.134  (0.289) |
| **Sr** |  |  |  |  |  |  |  |  |  |  |  |  |  | 0.259*  (0.037) | 0.305*  (0.013) |
| **V** |  |  |  |  |  |  |  |  |  |  |  |  |  |  | 0.313* (0.011) |
| **Zn** |  |  |  |  |  |  |  |  |  |  |  |  |  |  |  |

**Table S6.** Spearman correlation coefficients and p-value (in brackets) between bioaccessible metal(oid)s concentrations. Statistical significance represented by *** for p<0.001, ** for p<0.01, and *for p<0.05.

|  | **Al** | **Cr** | **Cu** | **Fe** | **Mn** | **Ni** | **V** | **Zn** |
| --- | --- | --- | --- | --- | --- | --- | --- | --- |
| **Al** |  | 0.101 (0.702) | 0.314* (0.024) | 0.416** (0.001) | 0.641** (0.000) | 0.201 (0.079) | 0.452** (0.000) | 0.138** (0.304) |
| **Cr** |  |  | -0.077 (0.812) | -0.105 (0.746) | 0.000 (1.000) | -0.400 (0.286) | 0.587 (0.366) | 0.285  (0.425) |
| **Cu** |  |  |  | 0.611** (0.000) | 0.501** (0.000) | 0.048  (0.767) | -0.015  (0.914) | 0.176  (0.284) |
| **Fe** |  |  |  |  | 0.876** (0.000) | 0.007  (0.966) | 0.350**  (0.004) | 0.199  (0.190) |
| **Mn** |  |  |  |  |  | -0.009  (0.527) | 0.525**  (0.000) | 0.325*  (0.030) |
| **Ni** |  |  |  |  |  |  | -0.243  (0.116) | -0.278  (0.153) |
| **V** |  |  |  |  |  |  |  | 0.074  (0.628) |
| **Zn** |  |  |  |  |  |  |  |  |

**Fig S1.** Compositions in percentages of major ions during 1-year sampling period and summer and winter seasons.

**Fig S2.** Compositions in percentages of metal(oid)s during 1-year sampling period and summer and winter seasons.

**Fig S3.** Compositions in percentages of PAHs during 1-year sampling period and summer and winter seasons.

**Fig. S4.** Air mass origin during 1-year sampling period (January 1^th^ – December 31^th^). *NA* North Atlantic, *NWA* Northwest Atlantic, *SWA* Southwest Atlantic, *WA* West Atlantic, *EU* Europe, *MED* Mediterranean, *NAF* North Africa, *RE* regional.

**Fig S5.** Compositions in percentages of metal(oid) inhalation bioaccessible concentrations during 1-year sampling period and summer and winter seasons.

**Fig S6.** Compositions in percentages of PAH inhalation bioaccessible concentrations during 1-year sampling period and summer and winter seasons.

**Fig. S7.** The air mass back trajectory analysis at 750, 1500 and 2500 m during Saharan dust incursion (15^th^ October 2017).


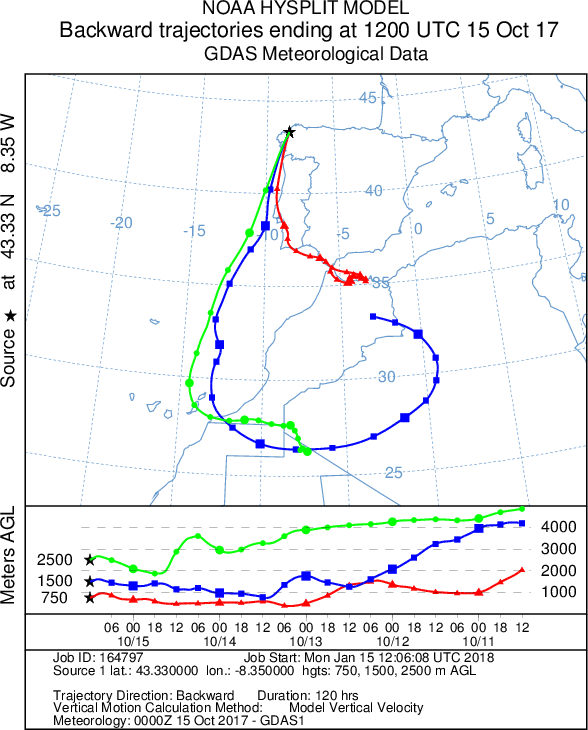


**References:**

Blanco–Heras GA, Turnes–Carou MI, López–Mahía P, Muniategui–Lorenzo S, Prada–Rodríguez D, Fernández–Fernández E (2008) Determination of organic anions in atmospheric aerosol samples by capillary electrophoresis after reversed pre–electrophoresis. Electrophoresis 29:1347–1354. <https://doi.org/10.1002/elps.200700413>

Colombo C, Monhemius AJ, Plant JA (2008) The estimation of the bioavailabilities of platinum, palladium and rhodium in vehicle exhaust catalysts and road dusts using a physiologically based extraction test. Sci Total Environ 389:46–51. https://doi.org/10.1016/jascitotenva2007.08.022

Fernández-Amado M, Prieto-Blanco MC, López-Mahía P, Muniategui-Lorenzo S, Prada-Rodríguez D (2016) A novel and cost-effective method for the determination of fifteen polycyclic aromatic hydrocarbons in low volume rainwater samples. Talanta 155:175–184. https://doi.org/10.1016/J.TALANTA.2016.04.032

Midander K, Pan J, Odnevall Wallinder I, Leygraf C (2007) Metal release from stainless steel particles in vitro—influence of particle size. J Environ Monit 9:74–81. https://doi.org/10.1039/B613919A

Moreda–Piñeiro J, Turnes–Carou I, Alonso–Rodríguez E, Moscoso–Pérez C, Blanco–Heras G, López–Mahía P, Muniategui–Lorenzo S, Prada–Rodríguez D (2015) The influence of oceanic air masses on concentration of major ions and trace metals in PM_2.5_ fraction at a coastal European suburban site. Water Air Soil Pollut 226:2240 https://doi.org/10.1007/S11270-014-2240-2

Pineiro-Iglesias M, Grueiro-Noche G, López-Mahía P, Muniategui-Lorenzo S, Prada-Rodríguez D (2004) Assessment of methodologies for airborne BaP analysis. Sci Total Environ 334–335:377–384. https://doi.org/10.1016/ j.scitotenv.2004.04.041

Pineiro-Iglesias M, López-Mahía P, Muniategui-Lorenzo S, Prada-Rodríguez D, Querol X, Alastuey A (2003) A new method for the simultaneous determination of PAH and metals in samples of atmospheric particulate matter. Atmos Environ 37:4171–4175. https://doi.org/10.1016/S1352-2310(03)00523-5

Sánchez–Piñero J, Moreda–Piñeiro J, Concha–Graña E, Fernández–Amado M, Muniategui–Lorenzo S, López–Mahía P (2021) Inhalation bioaccessibility estimation of polycyclic aromatic hydrocarbons from atmospheric particulate matter (PM_10_): Influence of PM_10_ composition and health risk assessment. Chemosphere 263:127847. https://doi.org/https://doi.org/10.1016/j.chemosphere.2020.127847
